# The effects of L-carnitine supplementation on cardiovascular risk factors in participants with impaired glucose tolerance and diabetes: a systematic review and dose–response meta-analysis

**DOI:** 10.1186/s13098-024-01415-8

**Published:** 2024-07-31

**Authors:** Rezvan Gheysari, Mahlagha Nikbaf-Shandiz, Amir Mehdi Hosseini, Niloufar Rasaei, Shabnam Hosseini, Hossein Bahari, Omid Asbaghi, Samira Rastgoo, Kian Goudarzi, Farideh Shiraseb, Reza Behmadi

**Affiliations:** 1 Shohada-E-Tajrish Hospital, Shahid Beheshti University of Medical Sciences, Tehran, Iran; 2 Student Research Committee, Tabriz University of Medical Sciences, Tabriz, Iran; 3 Faculty of Medical Sciences and Technologies, Science and Research Branch, Islamic Azad University, Tehran, Iran; 4 Department of Community Nutrition, School of Nutritional Sciences and Dietetics, Tehran University of Medical Sciences (TUMS), Tehran, Iran; 5 School of Human Nutrition, Faculty of Agricultural and Environmental Sciences, McGill University, Montreal, QC Canada; 6 Transplant Research Center, Clinical Research Institute, Mashhad University of Medical Sciences, Mashhad, Iran; 7 Cancer Research Center, Shahid Beheshti University of Medical Sciences, Tehran, Iran; 8 Student Research Committee, Shahid Beheshti University of Medical Sciences, Tehran, Iran; 9 Department of Cellular and Molecular Nutrition, Faculty of Nutrition Science and Food Technology, National Nutrition and Food Technology Research Institute, Shahid Beheshti University of Medical Sciences, Tehran, Iran; 10 Faculty of Medicine, Shahid Beheshti University of Medical Sciences, Tehran, Iran; 11 Department of Pediatrics, School of Medicine, Iran University of Medical Sciences, Tehran, Iran

**Keywords:** L-carnitine, Cardiovascular risk factors, Diabetes, Systematic review, Meta-analysis

## Abstract

**Aims:**

L-carnitine plays a role related to cardiometabolic factors, but its effectiveness and safety in CVD are still unknown. We aim to assess the effect of L-carnitine supplementation on CVD risk factors.

**Methods:**

A systematic literature search was conducted in PubMed, Web of Science, and Scopus until October 2022. The main outcomes were lipid profiles, anthropometric parameters, insulin resistance, serum glucose levels, leptin, blood pressure, and inflammatory markers. The pooled weighted mean difference (WMD) was calculated using a random-effects model.

**Results:**

We included the 21 RCTs (n = 2900) with 21 effect sizes in this study. L-carnitine supplementation had a significant effect on TG (WMD = − 13.50 mg/dl, p = 0.039), LDL (WMD = − 12.66 mg/dl, p < 0.001), FBG (WMD = − 6.24 mg/dl, p = 0.001), HbA1c (WMD = -0.37%, p = 0.013) HOMA-IR (WMD = -0.72, p = 0.038 (, CRP (WMD = − 0.07 mg/dl, P = 0.037), TNF-α (WMD = − 1.39 pg/ml, p = 0.033), weight (WMD = − 1.58 kg, p = 0.001 (, BMI (WMD = − 0.28 kg/m^2^, p = 0.017(, BFP (WMD = − 1.83, p < 0.001) and leptin (WMD = − 2.21 ng/ml, p = 0.003 (in intervention, compared to the placebo group, in the pooled analysis.

**Conclusions:**

This meta-analysis demonstrated that administration of L-carnitine in diabetic and glucose intolerance patients can significantly reduce TG, LDL-C, FBG, HbA1c, HOMA-IR, CRP, TNF-α, weight, BMI, BFP, and leptin levels.

PROSPERO registration code: CRD42022366992.

**Supplementary Information:**

The online version contains supplementary material available at 10.1186/s13098-024-01415-8.

## Introduction

Cardiovascular disease (CVD) continues to take the lives of approximately 19.7 million individuals worldwide annually. [1]. According to the World Health Organization (WHO), CVD encompasses coronary heart disease, cerebrovascular disease, rheumatic heart disease, and other related conditions [2, 3]. CVD is the cause of 50% of mortalities in people with type 2 diabetes (T2D) [4]. People with T2D hold an 8.8% share of the whole world’s population, and it is estimated to increase to 693 million people by 2045 [5]. Furthermore, it is crucial to note that the prevalence of individuals with prediabetes, a condition that can be reversed and independently heightens the likelihood of both T2D and CVD, is projected to escalate to approximately 587 million by the year 2045 [5]. In light of the considerable burden of CVD, it is essential and efficient to prioritize interventions aimed at modifying the risk factors of CVD in diabetic individuals. These interventions may include suggesting dietary modifications, increasing physical activity, reducing obesity and hypertension, as well as promoting smoking cessation [6]. In addition to examining dietary patterns and food groups, researchers have also focused on investigating the efficacy and safety of dietary supplements like L-carnitine in relation to cardiovascular disease. However, the findings regarding their effectiveness and safety in this context remain inconclusive [7].

L-carnitine, which is an active form of carnitine, plays a role in transporting long-chain fatty acids into the mitochondria. Previous literature has established a connection between L-carnitine and CVD [8]. The main sources of carnitine intake in humans are red meat and dairy products [9]. In 1999, Retter A. conducted a primary study that examined the role of carnitine in CVDs. The study's findings suggested that exogenous carnitine could potentially be used as an effective treatment for different cardiac diseases [10]. After that, many articles, but some previous review articles have assessed the effect of both endogenous [11] and exogenous L-carnitine on CVD risk factors [8, 12]. In a 2021 editorial authored by Pereira et al., the discovery of a recently identified adipokine called Chemerin was documented. This adipokine is found to play a crucial role in the initial phase of acute inflammation [13]. They showed that Chemerin concentration is decreased by the L-carnitine supplementation [13]. Koeth et al. conducted a study that demonstrated the role of trimethylamine-N-oxide (TMAO) in the body, specifically its association with L-carnitine (driver of L-carnitine) and its impact on the progression of T2D, CVD, and atherosclerosis [7]. Therefore, the result of the previous literature is still inconclusive regarding the effect of L-carnitine on CVD.

As far as our knowledge extends, no meta-analysis has been performed to examine the impact of L-carnitine on CVD risk factors in individuals with T2D or prediabetes. Consequently, this study intends to provide a comprehensive investigation into the effects of L-carnitine supplementation on various CVD risk factors, such as lipid profile, blood pressure, glycemic markers, inflammatory markers, and anthropometric and body composition measurements.

## Materials and methods

### Search strategy and study selection

The protocol for this study has been registered with PROSPERO and the findings are reported using the Preferred Reporting Items for Systematic Reviews and Meta-Analysis (PRISMA) approach. [14]. In order to gather relevant academic sources, a comprehensive search was carried out in reputable databases including PubMed, Web of Science, Scopus, and Cochrane. Specific search strategies tailored to each database were employed until October 2022. The search terms and keywords utilized were as follows: ("Vitamin BT" OR L-carnitine OR carnitine OR levocarnitine OR bicarnesine OR L-acetylcarnitine OR acetyl-L-carnitine) AND (Intervention OR "Intervention Study" OR "Intervention Studies" OR "controlled trial" OR randomized OR random OR randomly OR placebo OR "clinical trial" OR Trial OR "randomized controlled trial" OR "randomized clinical trial" OR RCT OR blinded OR "double blind" OR "double blinded" OR trial OR trials OR "Pragmatic Clinical Trial" OR "Cross-Over Studies" OR "Cross-Over" OR "Cross-Over Study" OR parallel OR "parallel study" OR "parallel trial"). In our study, there were no time and language restrictions in the advanced search strategy. To ensure comprehensive coverage, we checked all references of review articles, systematic reviews, meta-analyses and RCTs and other relevant studies to avoid missing studies.

Our search framework was as follows: Patients: Individuals with type 2 diabetes/prediabetes, impaired glucose tolerance, Intervention: Oral L-carnitine supplementation, Comparison: Non-L-carnitine supplementation as control or placebo group, Outcome: all CVD risk factors (lipid profile, blood pressure, glycemic markers, inflammatory markers, anthropometric and body composition measurements).

In our study, we considered several important criteria for the inclusion of clinical studies. These criteria included the requirement for clinical trials with a duration of at least one week, involving adult human subjects who were 18 years old or older. We also looked for studies that reported mean ± standard deviation (SD) or any effect size for outcomes that could be converted to mean and SD values. Additionally, we sought studies that evaluated the impact of L-carnitine supplementation on cardiovascular disease risk factors such as serum triglyceride (TG), total cholesterol (TC), low-density lipoprotein (LDL), high-density lipoprotein (HDL), fasting blood glucose, HbA1c, serum insulin, homeostasis model assessment-insulin resistance (HOMA-IR), systolic blood pressure (SBP), diastolic blood pressure (DBP), c-reactive protein (CRP), interleukin-6 (IL-6), tumor necrosis factor-alpha (TNF-α), apolipoprotein A (apo A), apolipoprotein B (apo B), weight, waist circumference (WC), body mass index (BMI), and body fat percentage (BFP).

We established exclusion criteria to ensure the validity and relevance of the selected studies: in-vivo or in-vitro studies, studies with children and adolescent participants, grey literature, conference abstracts, editorials, books, and RCTs without a control group.

### Data collection

(RG and OA) conducted title and abstract screening of studies. Any disagreements were resolved through discussion between them. RG and OA gathered information on various aspects including the first author's name, year of publication, country, type of clinical trial, and participant characteristics such as mean age, BMI, and sex. They also collected data on the duration of intervention, randomization, blinding, sample size, the number of participants in the intervention and control groups, form and dosage of supplemented L-carnitine, the health status of participants (impaired glucose tolerance, diabetic, pre-diabetic), as well as outcome values.

### Risk of bias assessment

Cochrane Collaboration tool [15] was used to screen for any biases such as random sequence generation, allocation concealment, participant and staff blindness, outcome assessor blinding, incomplete outcome data, selective reporting, and other biases (Table 1). (RG, OA) assessed the quality of each article and discussed any conflicting opinions.

**Table 1:** Risk of bias assessment

Study	Random sequence generation	Allocation concealment	Selective reporting	Other sources of bias	Blinding (participants and personnel)	Blinding (outcome assessment)	Incomplete outcome data	General risk of bias
Liang et al. 1998	U	H	H	H	L	U	L	Bad
Derosa et al. 2003	L	H	H	H	L	U	L	Bad
Rahbar et al. 2005	L	H	H	H	L	U	L	Bad
Solfrizzi et al. 2006	L	H	H	H	H	H	L	Bad
Santo et al. 2006	L	H	H	H	L	U	L	Bad
González-Ortiz et al. 2008	L	U	H	H	H	L	L	Bad
Malaguarnera et al. 2009	L	H	H	H	H	H	L	Bad
Galvano et al. 2009	L	H	H	L	L	U	L	Fair
Malaguarnera et al. 2009	L	H	H	H	H	H	L	Bad
Bloomer et al. 2009	L	H	H	H	L	U	L	Bad
Molfino et al. 2010	L	H	H	H	H	H	L	Bad
Derosa et al. 2011	L	H	H	L	L	U	L	Fair
Derosa et al. 2011	L	H	H	H	L	U	L	Bad
Barzegar et al. 2013	L	H	H	H	H	H	L	Bad
Ramazanpour et al. 2015	L	H	H	H	H	H	L	Bad
Ghorbani et al. 2017	L	H	H	H	L	U	L	Bad
Hassani and Ghorbani. 2018	L	H	H	H	L	U	L	Bad
Parvanova et al. 2018	L	L	H	L	L	U	L	Good
Bruls et al. 2019	L	H	H	H	L	U	L	Bad
El-Sheikh et al. 2019	L	L	H	L	L	U	L	Good
Talenezhad et al. 2020	L	L	H	L	L	U	L	Good

*U* unclear risk of bias, *L* low risk of bias, *H* high risk of bias

Good < 2 high risk of bias; Fair = 2 high risk of bias; Bad > 2 high risk of bias

### Certainty assessment

The study employed The Grading of Recommendations Assessment, Development, and Evaluation (GRADE) approach, which is designed to evaluate the level of certainty in the evidence presented [16].

### Statistical analyses

The data analysis was conducted using Stata version 11.0 (Stata Corp, College Station, TX). A significance level of < 0.05 was used for all tests, with two-tailed testing. The heterogeneity of evidence was assessed using the DerSimonian-Laird method to calculate the pooled weighted mean difference (WMD) [17]. Mean differences in outcomes between the control and intervention groups were computed in this study, comparing baseline measurements to post-intervention measurements. The SD of the mean difference was calculated using the following formula: SD = square root [(SD at baseline)^2^ + (SD at the end of study)^2^ − (2 r × SD at baseline × SD at the end of study)] [18]. To transform standard errors (SEs), 95 percent confidence intervals (CIs), or interquartile ranges (IQRs) into SDs, we used Hozo et al. approach: [SD = SE × √n (n = the number of individuals in each group)] [19]. For r, a correlation coefficient of 0.8 was used [20].

In order to investigate the source of variability, a subgroup analysis was conducted. The selection of subgroups was determined by the minimum number of studies required, as outlined in the criteria established by Fu et al. in 2011. For continuous variables, a minimum of 6 to 10 studies were necessary, while categorical subgroup variables required at least 4 studies [21, 22]. Subgroup analyses were performed regarding SBP (< 130 mmHg, ≥ 130 mmHg), DBP (< 80 mmHg, ≥ 80 mmHg), TG (< 150 mg/dl, ≥ 150 mg/dl), TC (< 200 mg/dl, ≥ 200 mg/dl), LDL-C (< 100 mg/dl, ≥ 100 mg/dl), HDL-C (< 40 mg/dl, ≥ 40 mg/dl), intervention duration (≤ 12 weeks, > 12 weeks), and dosage of L-carnitine (< 2 g/day, ≥ 2 g/day)., and baseline BMI [normal (18.5- 24.9 kg/m^2^), overweight (25–29.9 kg/m^2^) and obese (≥ 30 kg/m^2^)]. In the meta-analysis, the I^2^ or Cochrane’s Q test was used to measure statistical heterogeneity [23], with values greater than 40 percent indicating a strong heterogeneity [24]. Various methods, such as Begg's and Egger's tests, visual inspection of funnel plots, and others, were employed to evaluate the potential presence of publication bias [25, 26]. Sensitivity analyses were carried out to see how each study might affect the combined effect size. Trim-and-fill was employed to identify and mitigate the effects of publication bias [27]. Meta-regression was used to assess the possible impact of L-carnitine dosage and duration on CVD risk factors. Additionally, we conducted a dose–response analysis between L-carnitine supplementation and the variables under study using nonlinear regression.

## Results

### The flow of study selection

**Fig. 1:**
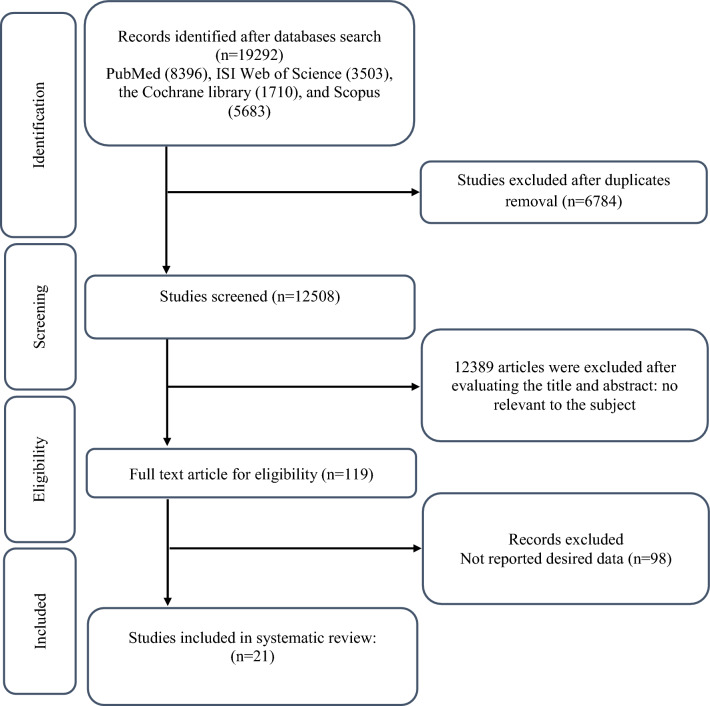
Flow chart of study selection for inclusion trials in the systematic review

**Fig. 2:**
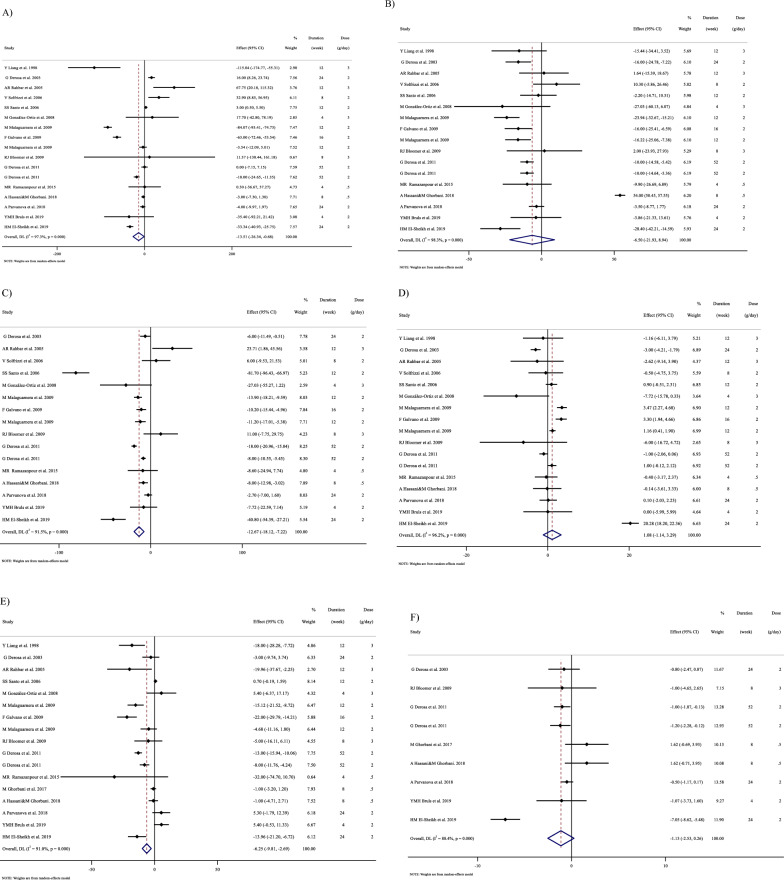

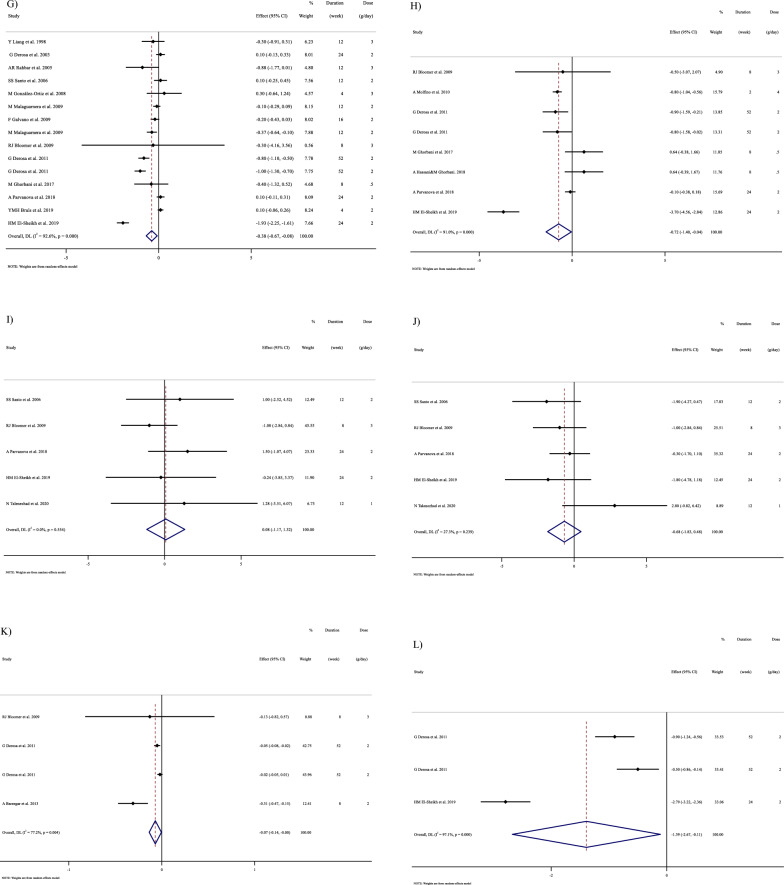

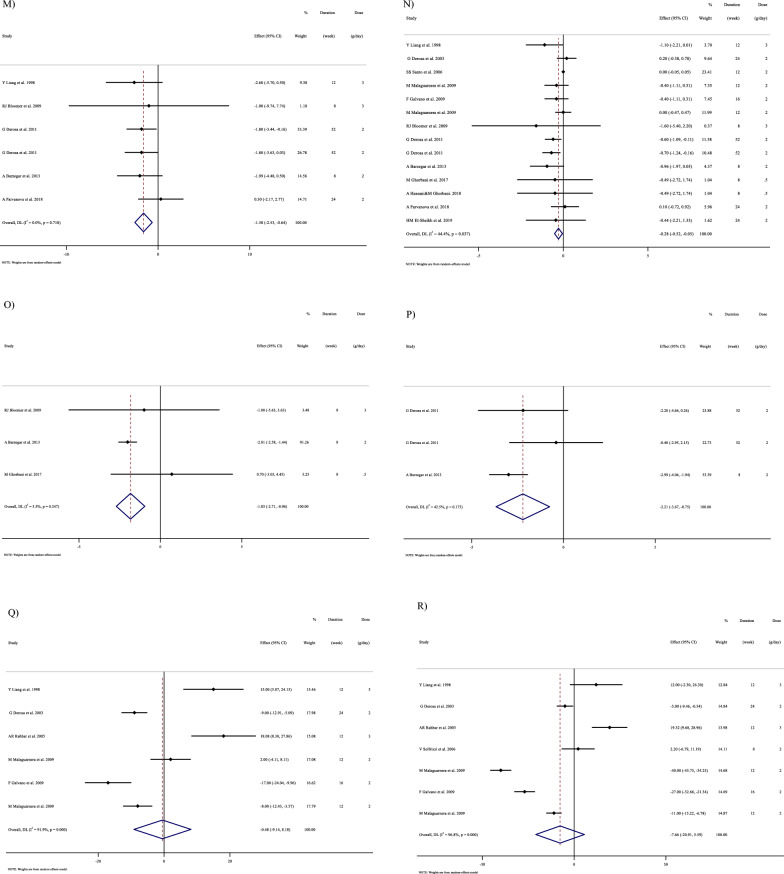
Forest plot detailing weighted mean difference and 95% confidence intervals (CIs) for the effect of carnitine consumption on **A** TG (mg/dl); **B** TC (mg/dl); **C** LDL (mg/dl); **D** HDL (mg/dl); **E** FBG (mg/dl); **F** Insulin (uU/ml); **G** HbA1c (%); **H** HOMA-IR; **I** SBP (mmHg); **J** DBP (mmHg); **K** CRP (mg/l); **L** TNF-α (pg/ml); M)weight (kg); **N** BMI (kg/m^2^); **O** BFP (%); **P** Leptin (ng/ml); **Q** Apo A (mg/dl) and **R** Apo B (mg/dl). *Apo A* Apolipoprotein A, *Apo B* Apolipoprotein B, *BFP* body fat percentage, *BMI* body mass index, *CI* confidence interval; *CRP* c-reactive protein, *FBG* fasting blood glucose, *HbA1c* hemoglobin A1c, *HDL* high-density lipoprotein, *HOMA-IR* homeostatic model assessment for insulin resistance, *LDL* low-density lipoprotein; DBP, diastolic blood pressure; *SBP* systolic blood pressure, *TC* total cholesterol, *TG* triglyceride, *TNF-α* tumor necrosis factor alpha, *WMD* weighted mean differences

In Fig. 1, we presented the flow chart outlining the study. The selection process and references obtained from the database are described in this figure. Initially, a total of 19,292 studies were identified through an electronic database search. We then excluded duplicated (n = 6784) and irrelevant studies (n = 12,508) based on titles and abstracts, leaving us with 119 full-text relevant articles to review. From these, 98 studies were excluded for reasons such as insufficient outcome data report, acute oral ingestion, or short duration of supplementation (< 1 week). Ultimately, 21 studies were included in the qualitative synthesis. Finally, we included a total of 21 studies [28–48]. The study design characteristics are shown in Supplementary Table 1. The WMD and 95% CI of TG, TC, LDL, HDL, FBG, insulin, HbA1c, HOMA-IR, SBP, DBP, CRP, TNF-α, weight, BMI, BFP, leptin, apo A and apo B and their changes are presented in Fig. 2A–R respectively. The studies included ranged from 1998 to 2020 years and originated in following countries: China (n = 1) [46], Italy (n = 10) [33–35, 37, 40–44, 47], Mexico (n = 1) [28], Iran (n = 6) [29, 30, 38, 39, 45, 48], Egypt (n = 1) [36], Netherlands (n = 1) [32] and USA (n = 1) [31]. The mean age ranged from 31 to 69.1 years and the baseline BMI of included studies ranged from 24.7 to 3446 kg/m^2^ in the intervention group, respectively. Four studies included only males or females [29, 38, 39, 48] and seventy studies included both sexes [28, 30–37, 40–47]. The supplementation duration of included studies ranged from 2 to 52 weeks. The daily dosage of L-carnitine supplementation ranged from 0. 5 to 4 g/day. Twenty parallel [28–31, 33–48] and one cross-over [32] studies were included in this study. Studies included diabetic patients [28–30, 33–41, 43–48], pre-diabetics [31], and impaired glucose tolerance patients [32, 42].

Out of the 21 RCTs, 17 studies have shown a significant reduction effect of L-carnitine supplementation on TG [28, 30, 32–38, 40, 43, 44, 46–48], 17 studies on TC [28, 30, 32–38, 40, 43, 44, 46–48], 16 studies on LDL [28, 30, 32–38, 40, 41, 43, 44, 47, 48], 17 studies on HDL [28, 30, 32–38, 40, 43, 44, 46–48], 17 studies on FBG [28, 30, 32–38, 40, 43, 44, 46–48], 9 studies on insulin [31–36, 38, 39, 43], 15 studies on HbA1c [28, 30, 32–37, 40, 43, 44, 46, 47], 8 studies on HOMA-IR [31, 34–36, 38, 39, 42, 43], 5 studies on SBP [31, 36, 43, 45, 47], 5 studies on DBP [31, 36, 43, 45, 47], 4 studies on CRP [29, 31, 34, 35], 3 studies on TNF-α [34–36], 6 studies on weight [29, 31, 34, 35, 43, 46], 14 studies on BMI [29, 31, 33–41, 43, 46, 47], 3 studies on BFP [29, 31, 39], 3 studies on leptin [29, 34, 35], 6 studies on apo A [30, 33, 37, 40, 41, 46] and 7 studies on apo B [30, 33, 37, 40, 41, 44, 46].

### Adverse events

Adverse effects were mentioned in the studies by Derosa et al. [34] (flatulence, constipation, abdominal pain, fatty/oily evacuation, increased defecation, fecal urgency, malaise) and Malaguarnera et al. [41] (gastrointestinal tract complaints).

### Qualitative data assessment

The qualitative data based on the Cochrane risk of bias assessment tool are presented in Table 1. Four studies had a low risk of bias [35, 36, 43, 45], six studies had a moderate risk of bias [31, 32, 34, 37–39] and eleven studies had a high risk of bias [28–30, 33, 40–42, 44, 46–48].

### Meta-analysis

#### Effect of L-carnitine supplementation on TG (mg/dl) and subgroup analysis

L-carnitine significantly affected TG (WMD = − 13.50 mg/dl, 95% CI − 26.33, − 0.67; P = 0.039; I^2^ = 97.3%, P < 0.001; Fig. 2A), according to the findings of a pooled analysis of 17 studies [17] with 3933 participants for TG [28, 30, 32–38, 40, 43, 44, 46–48]. L-carnitine consumption lowered TG (mg/dl) in baseline ≥ 150 mg/dl, (WMD = − 19.94 mg/dl, 95% CI − 38.79, − 1.10; P = 0.038), and in trial duration ≥ 12 weeks (WMD = − 19.83 mg/dl, 95% CI − 36.52, − 3.15; P = 0.020). Subgroup analyses indicated no significant between-study heterogeneity in all studies except in trial duration < 12 weeks (I^2^ = 0.0%, p = 0.892) and trial dose < 2 g/day (I^2^ = 50.4%, p = 0.073), were that probable sources of heterogeneity (Supplementary Table 2).

#### Effect of L-carnitine supplementation on TC (mg/dl) and subgroup analysis

L-carnitine did not reduce TC (WMD = − 6.49 mg/dl, 95% CI − 21.93, 8.93; P = 0.409; I^2^ = 98.3%, P < 0.001; Fig. 2B), according to the findings of a pooled analysis of 17 studies [17] with 3755 participants for TC [28, 30, 32–38, 40, 43, 44, 46–48].

L-carnitine consumption lowered TC (mg/dl) in baseline ≥ 200 mg/dl (WMD = − 12.82 mg/dl, 95% CI − 17.45, − 8.19; P < 0.001), and in trial duration ≥ 12 weeks (WMD = -12.52 mg/dl, 95% CI − 16.95, − 8.10; P < 0.001). Also, L-carnitine consumption lowered TG (mg/dl) in intervention dose ≥ 2 g/day (WMD = -11.02 mg/dl, 95% CI − 15.37, − 6.68; P < 0.001), in addition, in obese (> 30 kg/m^2^), (WMD = − 9.38 mg/dl, 95% CI − 14.82, − 3.94; P = 0.001). Subgroup analyses indicated no significant between-study heterogeneity in all studies (Supplementary Table 2).

#### Effect of L-carnitine supplementation on LDL-C (mg/dl) and subgroup analysis

L-carnitine significantly affected LDL-C (WMD = − 12.66 mg/dl, 95% CI − 18.12, − 7.21; P < 0.001; I^2^ = 91.5%, P < 0.001; Fig. 2C), according to the findings of a pooled analysis of 16 studies [16] with 3647 participants for LDL-C [28, 30, 32–38, 40, 41, 43, 44, 47, 48]. L-carnitine consumption lowered LDL-C (mg/dl) in baseline ≥ 100 mg/dl, (WMD = − 15.03 mg/dl, 95% CI − 20.78, − 9.28; P < 0.001), and in trial duration ≥ 12 weeks (WMD = − 16.17 mg/dl, 95% CI − 22.97, − 9.36 P < 0.001). Also, L-carnitine consumption lowered LDL-C (mg/dl) in any intervention dose (< 2 and ≥ 2 g/day) (WMD = -0.05 mg/dl, 95%CI: -12.81, -3.28; P = 0.001) and (WMD = -13.34 mg/dl, 95%CI: -19.45, -7.23; P < 0.001), respectively. In addition, in any baseline of BMI (overweight (25–29.9 kg/m^2^) and obese (> 30 kg/m^2^)), (WMD = − 7.23 mg/dl, 95% CI − 11.59, − 2.86; P = 0.001) and (WMD = − 27.17 mg/dl, 95% CI − 39.38, − 14.96; P < 0.001) respectively. Subgroup analyses indicated no significant between-study heterogeneity in all studies except in trial duration < 12 weeks (I^2^ = 39.8%, p = 0.140) and trial dose < 2 g/day (I^2^ = 0.0%, p = 0.945), were that probable sources of heterogeneity (Supplementary Table 2).

#### Effect of L-carnitine supplementation on HDL (mg/dl) and subgroup analysis

L-carnitine did not significantly affect HDL (WMD = 1.07 mg/dl, 95% CI − 1.13, 3.28; P = 0.341; I^2^ = 96.2%, P < 0.001; Fig. 2D), according to the findings of a pooled analysis of 17 studies [17] with 3487 participants for HDL [28, 30, 32–38, 40, 43, 44, 46–48]. Subgroup analyses conducted have shown that L-carnitine supplementation had not any significant effect in all subgroups (Supplementary 2). Subgroup analyses indicated no significant between-study heterogeneity in all studies except in trial duration < 12 weeks (I^2^ = 0.0%, p = 0.543) and trial dose < 2 g/day (I^2^ = 0.0%, p = 0.909), were that probable sources of heterogeneity (Supplementary Table 2).

#### Effect of L-carnitine supplementation on FBG (mg/dl) and subgroup analysis

17 effect sizes from 17 studies (n total = 2640) [28, 30, 32–38, 40, 43, 44, 46–48]., have shown L-carnitine supplementation had a significant effect on FBG (mg/dl) in intervention, compared to a placebo (WMD = -6.24 mg/dl, 95% CI − 9.80, − 2.69; P = 0.001; I^2^ = 91.0%, P < 0.001; Fig. 2E). L-carnitine consumption lowered FBG (mg/dl) in trial duration ≥ 12 weeks (WMD = -9.44 mg/dl, 95%CI: -14.92, -3.96; P = 0.001). Also, L-carnitine consumption lowered FBG (mg/dl) in intervention dose ≥ 2 g/day (WMD = − 7.14 mg/dl, 95% CI − 11.86, − 2.41; P = 0.003), in addition, in overweight (BMI 25–29.9 kg/m^2^) (WMD = − 6.29 mg/dl, 95% CI − 10.96, − 1.62; P = 0.008). Subgroup analyses indicated no significant between-study heterogeneity in all studies except in trial duration < 12 weeks (I^2^ = 35.5%, p = 0.170) and trial dose < 2 g/day (I^2^ = 1.0%, p = 0.364), were probable sources of heterogeneity (Supplementary Table 2).

#### Effect of L-carnitine supplementation on insulin (uU/ml) and subgroup analysis

9 effect sizes from 9 studies (n total = 1646) [31–36, 38, 39, 43], have shown L-carnitine supplementation had not a significant effect on insulin (uU/ml) in intervention, compared to a placebo (WMD = − 1.131 uU/ml, 95% CI − 2.52, 0.26; P = 0.112; I^2^ = 88.4%, P < 0.001; Fig. 2F). L-carnitine consumption lowered insulin in trial duration ≥ 12 weeks (WMD = − 2.03 uU/ml, 95% CI − 3.83, -0.24 P = 0.026). Also, L-carnitine consumption lowered insulin in intervention dose ≥ 2 g/day (WMD = − 1.82 uU/ml, 95% CI − 3.35, − 0.30; P = 0.019), in addition, in obese (BMI > 30 kg/m^2^) (WMD = − 2.33 uU/ml, 95% CI − 4.45, − 0.20; P = 0.031). Subgroup analyses indicated no significant between-study heterogeneity in all studies except in trial duration < 12 weeks (I^2^ = 19.5%, p = 0.292) and trial dose < 2 g/day (I^2^ = 0.0%, p = 1.000), and in overweight patients (I^2^ = 27.5%, p = 0.239) were that probable sources of heterogeneity (Supplementary Table 2).

#### Effect of L-carnitine supplementation on serum HbA1c (%) and subgroup analysis

There were 15 effect sizes from 15 studies (n total = 1626) [28, 30, 32–37, 40, 43, 44, 46, 47], that have shown L-carnitine supplementation had a significant effect on HbA1c (%) (WMD = − 0.37%, 95% CI − 0.67, − 0.07; P = 0.013; I^2^ = 92.6%, P < 0.001; Fig. 2G). Subgroup analyses have shown that L-carnitine supplementation had a reduction effect on HbA1c trial duration ≥ 12 weeks (WMD = − 0.46%, 95% CI − 0.81, − 0.11; P = 0.010). Subgroup analyses indicated no significant between-study heterogeneity in all studies except in trial duration < 12 weeks (I^2^ = 0.0%, p = 0.720) was a probable source of heterogeneity (Supplementary Table 2).

#### Effect of L-carnitine supplementation on HOMA-IR and subgroup analysis

L-carnitine supplementation had a significant effect on HOMA-IR (WMD = − 0.72, 95% CI − 1.40, − 0.04; P = 0.038; I^2^ = 91.0%, P < 0.001; Fig. 2H), with 8 effect sizes from 8 studies (n total = 1827) [31, 34–36, 38, 39, 42, 43]. Subgroup analyses have shown that L-carnitine supplementation had a reduction effect on HOMA-IR in trial dose ≥ 2 g/day (WMD = − 1.14, 95%CI: − 1.90, − 0.37; P = 0.004). Subgroup analyses indicated no significant between-study heterogeneity in all studies except in trial dose < 2g/day (I^2^ = 0.0%, p = 1.000) was a probable source of heterogeneity (Supplementary Table 2).

#### Effect of L-carnitine supplementation on SBP (mmHg) and subgroup analysis

In total, 5 effect sizes from 5 trials were considered in this analysis, representing a population of 1412 people [31, 36, 43, 45, 47]. After consuming L-carnitine, pooled effect sizes did not show a substantial drop in SBP (WMD = 0.07 mmHg, 95% CI: − 1.16, 1.32; P = 0.904; I^2^ = 0.0%, P = 0.554; Fig. 2I). Subgroup analyses have shown that L-carnitine supplementation had not a reduction effect on SBP in any subgroup. Subgroup analyses indicated significant between-study heterogeneity in all studies were probable sources of heterogeneity (Supplementary Table 2).

#### Effect of L-carnitine supplementation on DBP (mmHg) and subgroup analysis

L-carnitine supplementation had not a significant effect on DBP (WMD = -0.67 mmHg, 95% CI − 1.82, 0.47; P = 0.251; I^2^ = 27.3%, P = 0.239; Fig. 2J), with 5 effect sizes from 5 studies (n total = 1254) [31, 36, 43, 45, 47]. Subgroup analyses have shown that L-carnitine supplementation had not a reduction effect on SBP in any subgroup. Subgroup analyses indicated significant between-study heterogeneity in all studies were probable sources of heterogeneity (Supplementary Table 2).

#### Effect of L-carnitine supplementation on CRP (mg/dl) and subgroup analysis

4 effect sizes from 4 studies (n total = 1779) [29, 31, 34, 35], have shown L-carnitine supplementation had a significant effect on CRP (mg/dl) in intervention, compared to a placebo (WMD = -0.07 mg/dl, 95%CI: -0.13, -0.01; P = 0.037; I^2^ = 77.2%, P = 0.004; Fig. 2K). L-carnitine consumption lowered CRP in any trial duration (< 12 and ≥ 12 weeks), (WMD = − 0.30 mg/dl, 95% CI − 0.45, − 0.14; P < 0.001) and (WMD = -0.03 mg/dl, 95% CI − 0.06, − 0.01; P = 0.024) respectively. Subgroup analyses indicated significant between-study heterogeneity in all studies were probable sources of heterogeneity (Supplementary Table 2).

#### Effect of L-carnitine supplementation on TNF-α (pg/ml) and subgroup analysis

3 effect sizes from 3 studies (n total = 905) [34–36], have shown L-carnitine supplementation had a significant effect on TNF-α (pg/ml) in intervention, compared to a placebo (WMD = − 1.39 pg/ml, 95%CI: − 2.67, − 0.11; P = 0.033; I^2^ = 97.1%, P < 0.001; Fig. 2L) (Supplementary Table 2).

#### Effect of L-carnitine supplementation on weight (kg) and subgroup analysis

L-carnitine supplementation had a significant effect on weight (WMD = − 1.58 kg, 95% CI − 2.53, − 0.63; P = 0.001; I^2^ = 0.0%, P = 0.718; Fig. 2M), with 6 effect sizes from 6 studies (n total = 2641) [29, 31, 34, 35, 43, 46]. L-carnitine consumption lowered weight in trial duration ≥ 12 weeks (WMD = − 1.52 kg, 95% CI − 2.55, − 0.48; P = 0.004). Also, L-carnitine consumption lowered insulin in obese (BMI > 30 kg/m^2^) (WMD = − 1.48 kg, 95% CI − 2.48, − 0.48; P = 0.004). Subgroup analyses indicated significant between-study heterogeneity in all studies were probable sources of heterogeneity (Supplementary Table 2).

#### Effect of L-carnitine supplementation on BMI (kg/m^2^) and subgroup analysis

L-carnitine supplementation had a significant effect on BMI (WMD = − 0.28 kg/m^2^, 95% CI − 0.51, − 0.05; P = 0.017; I^2^ = 44.4%, P = 0.037; Fig. 2N), with 14 effect sizes from 14 studies (n total = 3815) [29, 31, 33–41, 43, 46, 47]. L-carnitine consumption lowered BMI in trial duration < 12 weeks (WMD = -0.86 kg/m^2^, 95% CI − 1.68, − 0.03; P = 0.041). Subgroup analyses indicated significant between-study heterogeneity in trial duration < 12 weeks (I^2^ = 0.0%, p = 0.941) and overweight patients (I^2^ = 0.0%, p = 0.507) were probable sources of heterogeneity.

#### Effect of L-carnitine supplementation on BFP (%) and subgroup analysis

L-carnitine supplementation had a significant effect on BFP (WMD = -1.83%, 95% CI − 2.70, − 0.95; P < 0.001; I^2^ = 5.5%, P = 0.347; Fig. 2O), with 3 effect sizes from 3 studies (n total = 507) [29, 31, 39].

#### Effect of L-carnitine supplementation on leptin (ng/ml) and subgroup analysis

L-carnitine supplementation had a significant effect on leptin (ng/ml) (WMD = − 2.21 ng/ml, 95% CI − 3.67, − 0.75; P = 0.003; I^2^ = 42.5%, P = 0.175; Fig. 2P), with 3 effect sizes from 3 studies (n total = 647) [29, 34, 35].

#### Effect of L-carnitine supplementation on apo A (mg/dl) and subgroup analysis

L-carnitine supplementation had no significant effect on apo A (mg/dl) (WMD = -0.48 mg/dl, 95% CI − 9.14, 8.13; P = 0.913; I^2^ = 91.9%, P < 0.001; Fig. 2Q), with 6 effect sizes from 6 studies (n total = 642) [30, 33, 37, 40, 41, 46].

#### Effect of L-carnitine supplementation on apo B (mg/dl) and subgroup analysis

L-carnitine supplementation had no significant effect on apo B (mg/dl) (WMD = − 7.66 mg/dl, 95% CI − 20.91, 5.58; P = 0.257; I^2^ = 96.8%, P < 0.001; Fig. 2R), with 7 effect sizes from 7 studies (n total = 694) [30, 33, 37, 40, 41, 44, 46].

### Nonlinear dose–response analysis

**Fig. 3:**
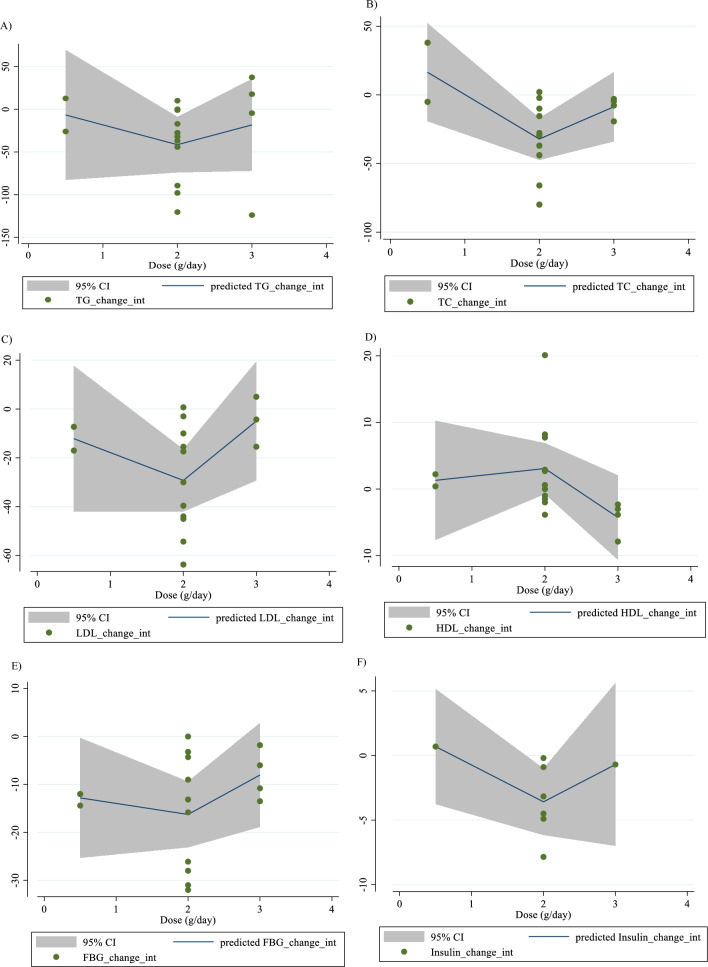

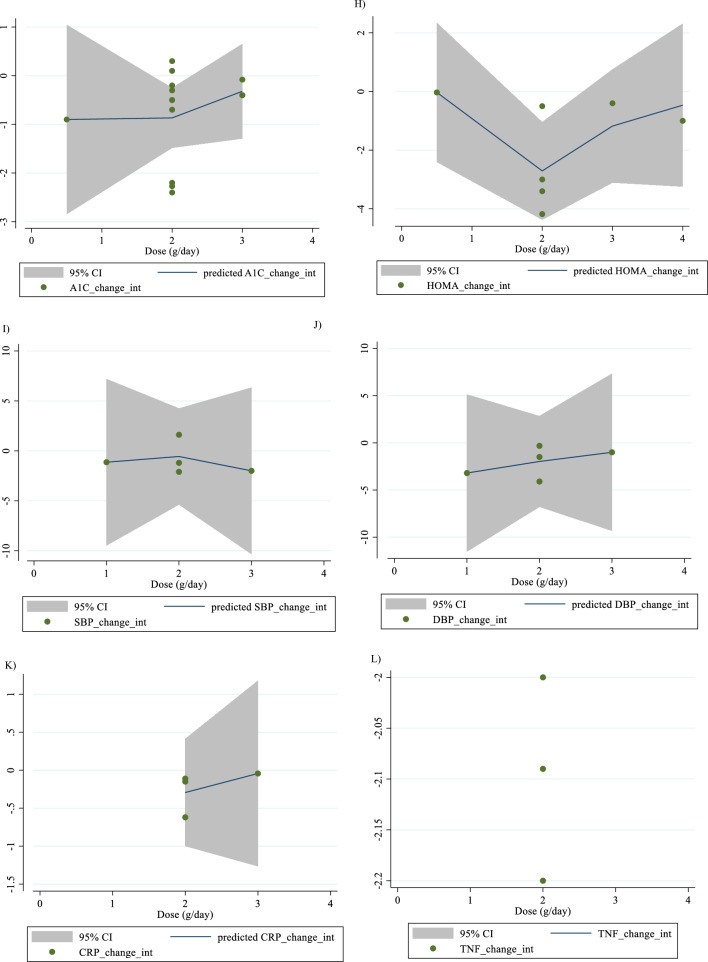

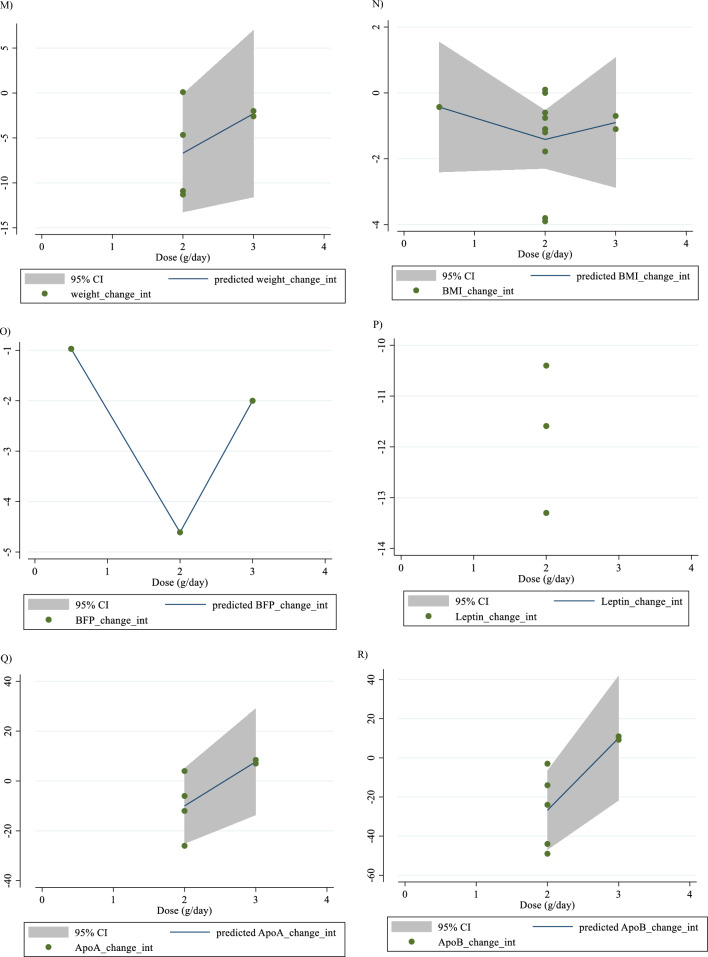
Non-linear dose–response relations between carnitine consumption and absolute mean differences. Dose–response relations between dose (mg/day) and absolute mean differences in **A** TG (mg/dl); **B** TC (mg/dl); **C** LDL (mg/dl); **D** HDL (mg/dl); **E** FBG (mg/dl); **F** Insulin (uU/ml); **G** HbA1c (%); **H** HOMA-IR; **I** SBP (mmHg); **J** DBP (mmHg); K) CRP (mg/l); **L** TNF-α (pg/ml); **M** weight (kg); **N** BMI (kg/m^2^); **O** BFP (%); **P** Leptin (ng/ml); **Q** Apo A (mg/dl) and **R** Apo B (mg/dl). *Apo A* Apolipoprotein A, *Apo B* Apolipoprotein B, *BFP* body fat percentage, *BMI* body mass index, *CI* confidence interval, *CRP* c-reactive protein, *FBG* fasting blood glucose, *HbA1c* hemoglobin A1c, *HDL* high-density lipoprotein, *HOMA-IR* homeostatic model assessment for insulin resistance, *LDL* low-density lipoprotein; DBP, diastolic blood pressure, *SBP* systolic blood pressure, *TC* total cholesterol, *TG* triglyceride; *TNF-α* tumor necrosis factor alpha, *WMD* weighted mean differences

**Fig. 4:**
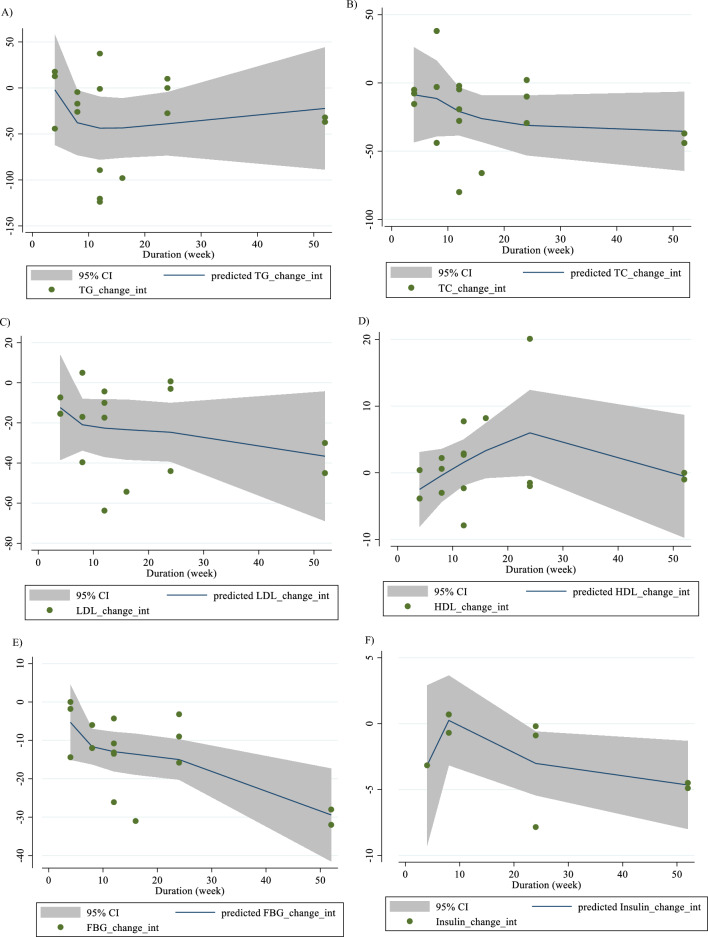

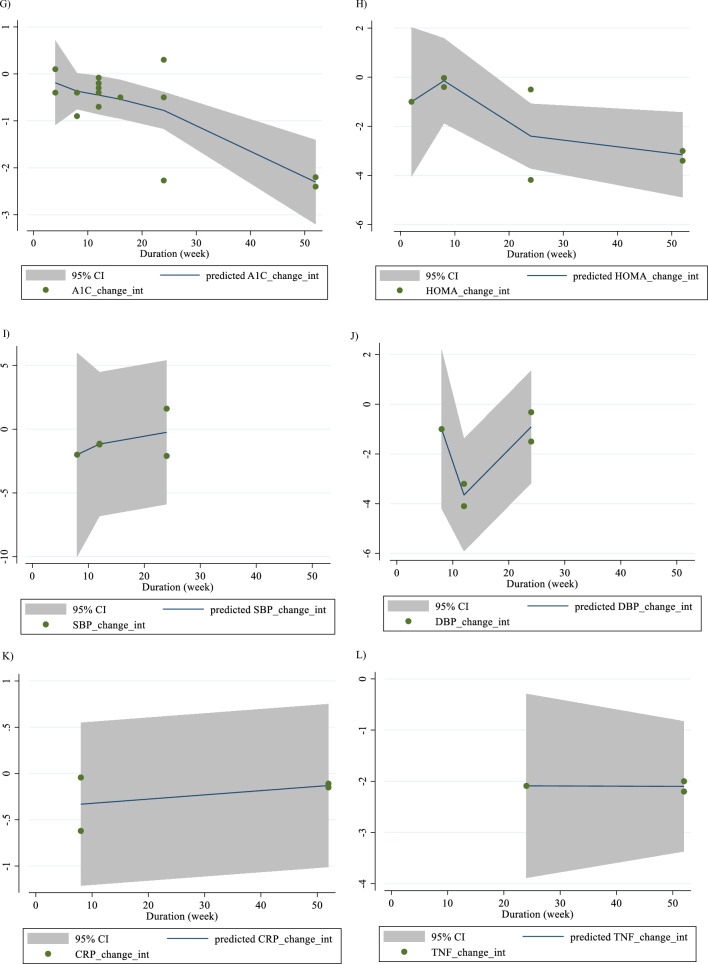

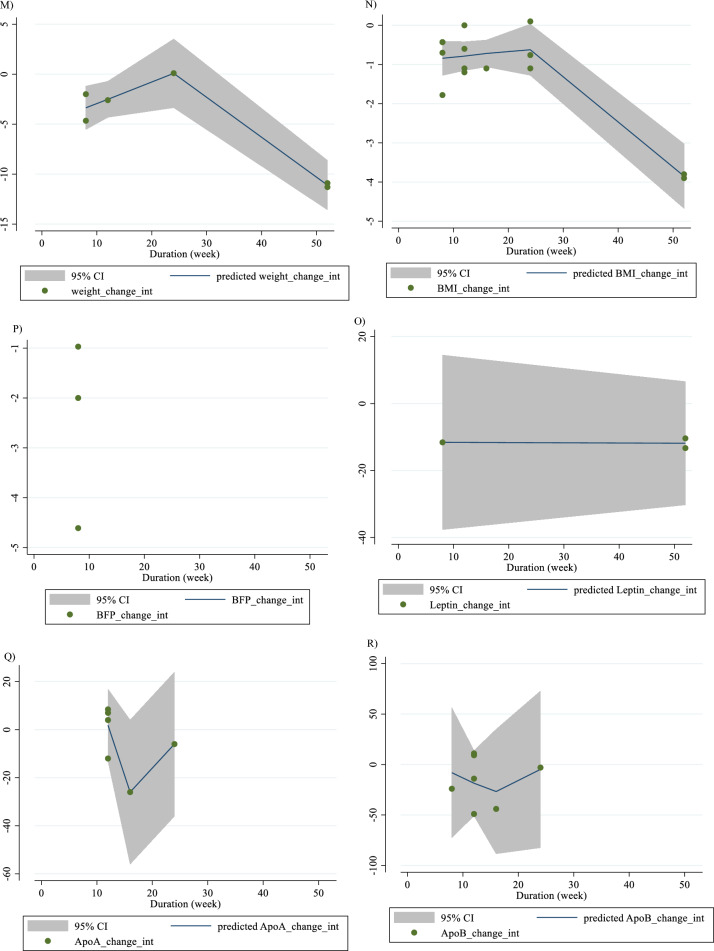
Non-linear dose–response relations between carnitine consumption and absolute mean differences. Dose–response relations between duration of intervention (week) and absolute mean differences in **A** TG (mg/dl); **B** TC (mg/dl); **C** LDL (mg/dl); **D** HDL (mg/dl); **E** FBG (mg/dl); **F** Insulin (uU/ml); **G** HbA1c (%); **H** HOMA-IR; I) SBP (mmHg); **J** DBP (mmHg); K) CRP (mg/l); **L** TNF-α (pg/ml); **M** weight (kg); **N** BMI (kg/m^2^); **O** BFP (%); P) Leptin (ng/ml); **Q** Apo A (mg/dl) and R) Apo B (mg/dl). *Apo A* Apolipoprotein A, *Apo B* Apolipoprotein B, *BFP* body fat percentage, *BMI* body mass index, *CI* confidence interval, *CRP* c-reactive protein, *FBG* fasting blood glucose, *HbA1c* hemoglobin A1c; HDL, high-density lipoprotein; *HOMA-IR* homeostatic model assessment for insulin resistance; *LDL* low-density lipoprotein; *DBP* diastolic blood pressure, *SBP* systolic blood pressure, *TC* total cholesterol, *TG* triglyceride, *TNF-α* tumor necrosis factor alpha, *WMD* weighted mean differences

For the dose–response analysis between L-carnitine supplementation and TG, TC, LDL, HDL, FBG, insulin, HbA1c, HOMA-IR, SBP, DBP, CRP, TNF-α, weight, BMI, BFP, leptin, apo A and apo B, we used a one-stage nonlinear dose–response analysis. There was no significant nonlinear relationship between dose (g/day) (coefficients =− 110.61, p = 0.451) and duration of intervention (weeks) (coefficients = − 146.67, p = 0.408) and changes in TG (Figs. 3A and 4A). Also, there was no significant nonlinear relationship between dose (g/day) (coefficients = 110.78, p = 0.419) and duration of intervention (weeks) (coefficients = − 146.67, p = 0.408) and changes in TC (Figs. 3B and 4B). In addition, there was no significant nonlinear relationship between dose (g/day) (coefficients = − 117.75, p = 0.080) and duration of intervention (weeks) (coefficients = 1.81, p = 0.483) and changes in LDL (Figs. 3C and 4C). Also, there was no significant nonlinear relationship between dose (g/day) (coefficients = 35.84, p = 0.051) and duration of intervention (weeks) (coefficients = 50.40, p = 0.085) and changes in HDL (Figs. 3D and 4D). Also, there was no significant nonlinear relationship between dose (g/day) (coefficients = − 40.03, p = 0.191) and changes in FBG, although there was a significant linear relationship between duration of the intervention (coefficients = − 0.11, p = 0.029) and changes in FBG (Figs. 3E and 4E). The optimum duration of supplementation near 50 weeks has shown a prominent effect on the decrement of FBG. We did not find a significant nonlinear relationship between dose (g/day) (coefficients = -13.77, p = 0.349) and duration of intervention (weeks) (coefficients = − 3.19, p = 0.087) and changes in insulin (Figs. 3F and 4F). Also, there was no significant nonlinear relationship between dose (g/day) (coefficients = -2.68, p = 0.317) and changes in HbA1c, although there was a significant linear relationship between the duration of the intervention (coefficients = -0.07, p = 0.003) and changes in HbA1c (Figs. 3G and 4G). The effective duration of supplementation near 50 weeks has shown a prominent effect on decreasing HbA1c. Also, there was no significant nonlinear relationship between dose (g/day) (coefficients = − 7.26, p = 0.156) and changes in HOMA-IR, although there was a significant linear relationship between the duration of the intervention (coefficients = 3.77, p = 0.032) and changes in HOMA-IR (Figs. 3H and 4H). The optimum duration of supplementation near 50 weeks has shown a prominent effect on the decrement of HOMA-IR. We did not find a significant nonlinear relationship between dose (g/day) (coefficients = − 8.75, p = 0.590) and duration of intervention (weeks) (coefficients = − 1.85, p = 0.690) and changes in SBP (Figs. 3I and 4I). We did not find a significant nonlinear relationship between dose (g/day) (coefficients = − 3.70, p = 0.600) and duration of intervention (weeks) (coefficients = 15.52, p = 0.060) and changes in DBP (Figs. 3J and 4J). Also, we did not find a significant nonlinear relationship between dose (g/day) (coefficients = − 1.80, p = 0.524) and duration of intervention (weeks) (coefficients = − 0.13, p = 0.558) and changes in CRP (Figs. 3K and 4K). We did not find a significant nonlinear relationship between the duration of the intervention (weeks) (coefficients = 0.07, p = 0.963) and changes in TNF-α (Figs. 3L and 4L). Also, there was no significant nonlinear relationship between dose (g/day) (coefficients = − 31.60, p = 0.344) and weight changes, although there was a significant linear relationship between duration of the intervention (coefficients = 1.60, p = 0.049) and changes in weight (Figs. 3M and 4M). Also, we did not find a significant nonlinear relationship between dose (g/day) (coefficients = − 0.98, p = 0.310) and duration of the intervention (weeks) (coefficients = 0.06, p = 0.340 and changes in BMI (Figs. 3N and 4N). That seems a duration of supplementation ≥  25 weeks has a decreasing effect on weight. We did not find a significant nonlinear relationship between the duration of the intervention (weeks) (coefficients = 0.17, p = 0.934) and changes in leptin (Figs. 3P and 4P). There was no significant nonlinear relationship between dose (g/day) (coefficients = − 127.58, p = 0.133) and duration of intervention (weeks) (coefficients = − 11.79, p = 0.698) and changes in apo A (Figs. 3Q and 4Q). Also, there was no significant nonlinear relationship between dose (g/day) (coefficients = − 265.78, p = 0.053) and duration of intervention (weeks) (coefficients = − 14.11, p = 0.627) and changes in apo B (Figs. 3R and 4R).

### Meta-regression analysis

**Fig. 5:**
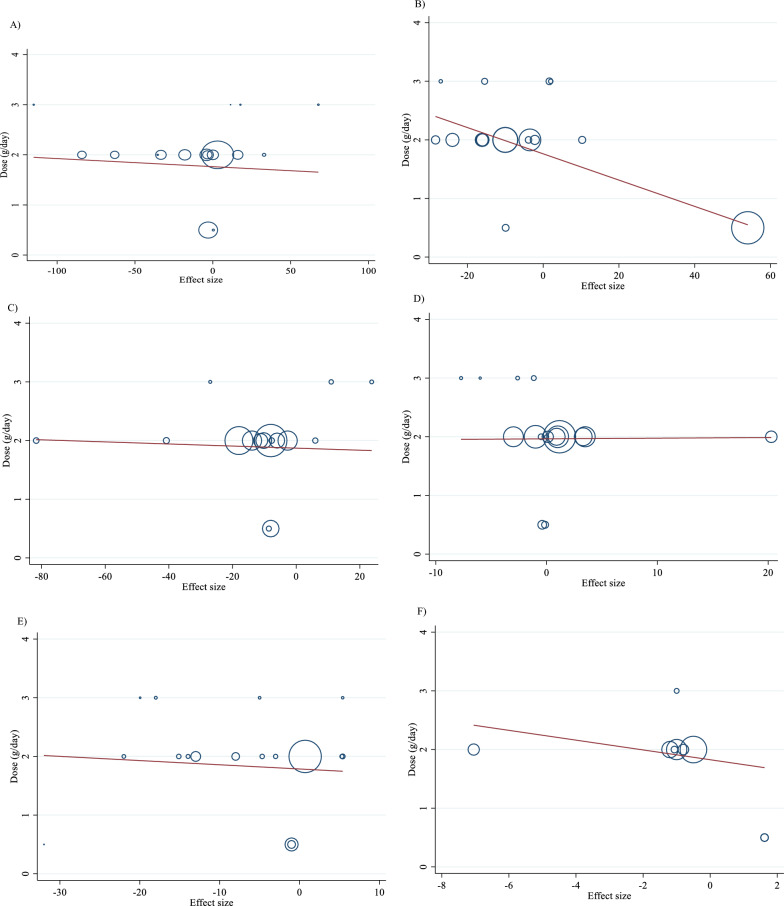

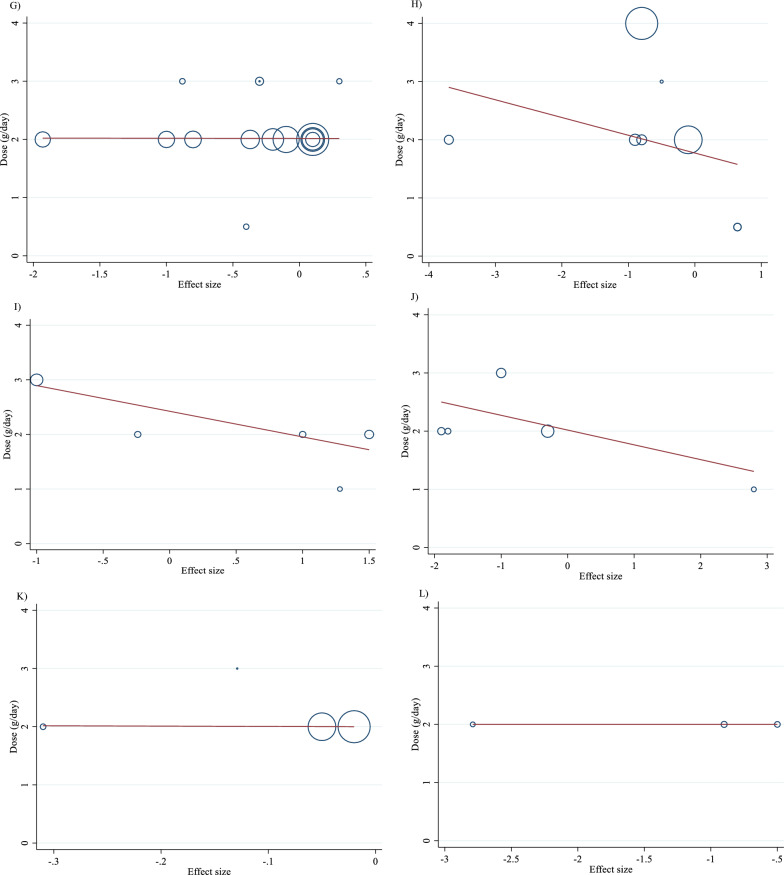

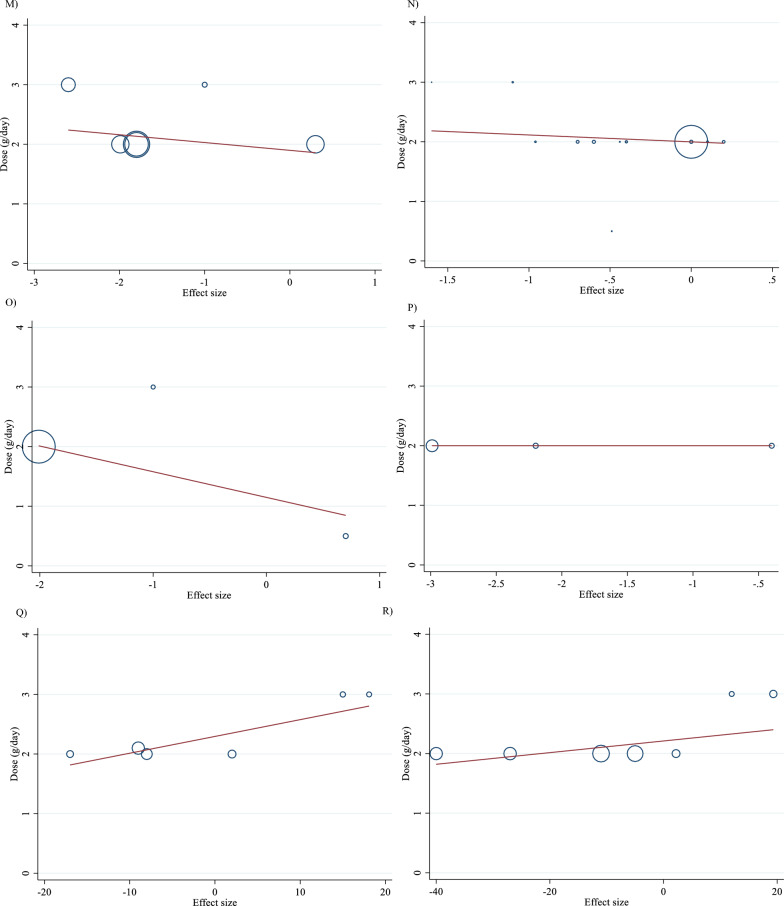
Linear dose–response relations between carnitine consumption and absolute mean differences. Dose–response relations between dose (mg/day) and absolute mean differences in **A** TG (mg/dl); **B** TC (mg/dl); **C** LDL (mg/dl); **D** HDL (mg/dl); **E** FBG (mg/dl); **F** Insulin (uU/ml); **G** HbA1c (%); **H** HOMA-IR; **I** SBP (mmHg); **J** DBP (mmHg); **K** CRP (mg/l); **L** TNF-α (pg/ml); **M** weight (kg); **N** BMI (kg/m^2^); **O** BFP (%); **P** Leptin (ng/ml); Q) Apo A (mg/dl) and **R** Apo B (mg/dl). *Apo A* Apolipoprotein A, *Apo B* Apolipoprotein B, *BFP* body fat percentage; *BMI* body mass index; *CI* confidence interval, *CRP* c-reactive protein; *FBG* fasting blood glucose, *HbA1c* hemoglobin A1c, *HDL* high-density lipoprotein; *HOMA-IR* homeostatic model assessment for insulin resistance, *LDL* low-density lipoprotein, *DBP* diastolic blood pressure, *SBP* systolic blood pressure, *TC* total cholesterol, *TG* triglyceride, *TNF-α* tumor necrosis factor alpha, *WMD* weighted mean differences

**Fig. 6:**
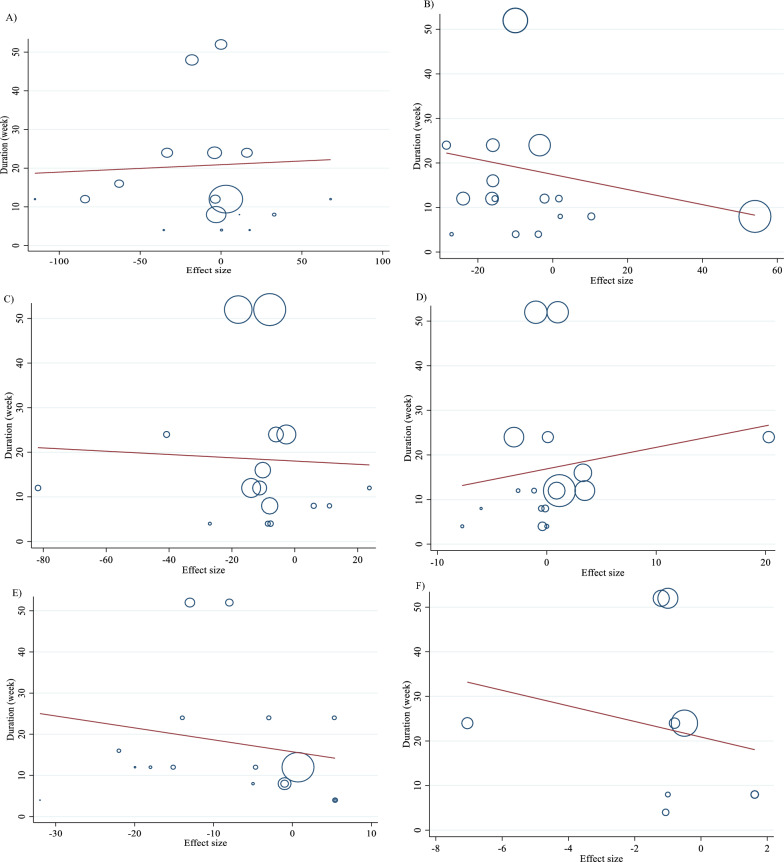

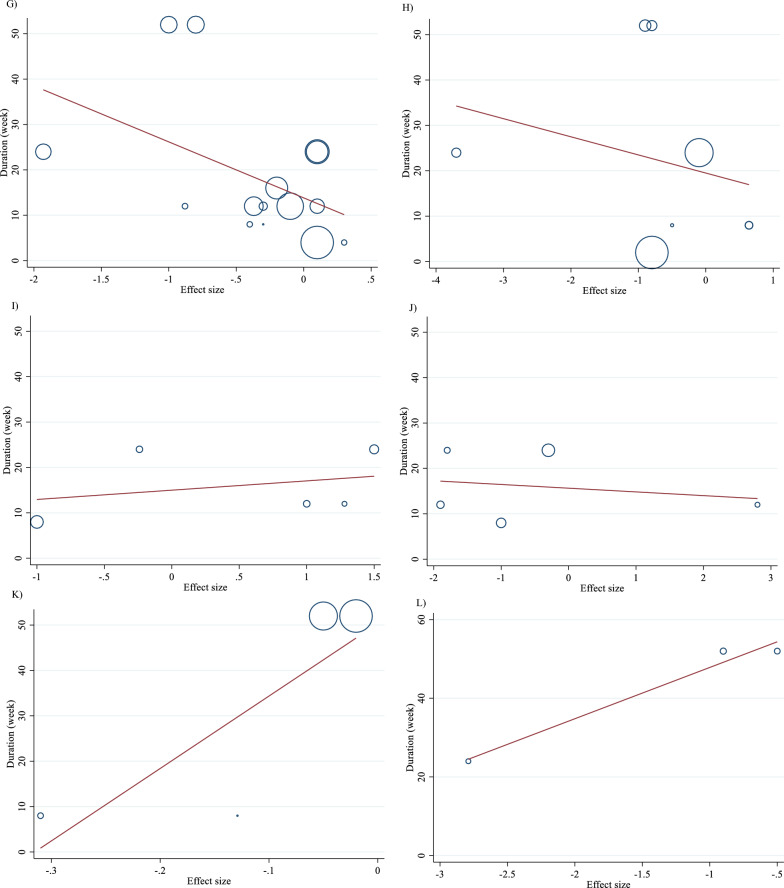

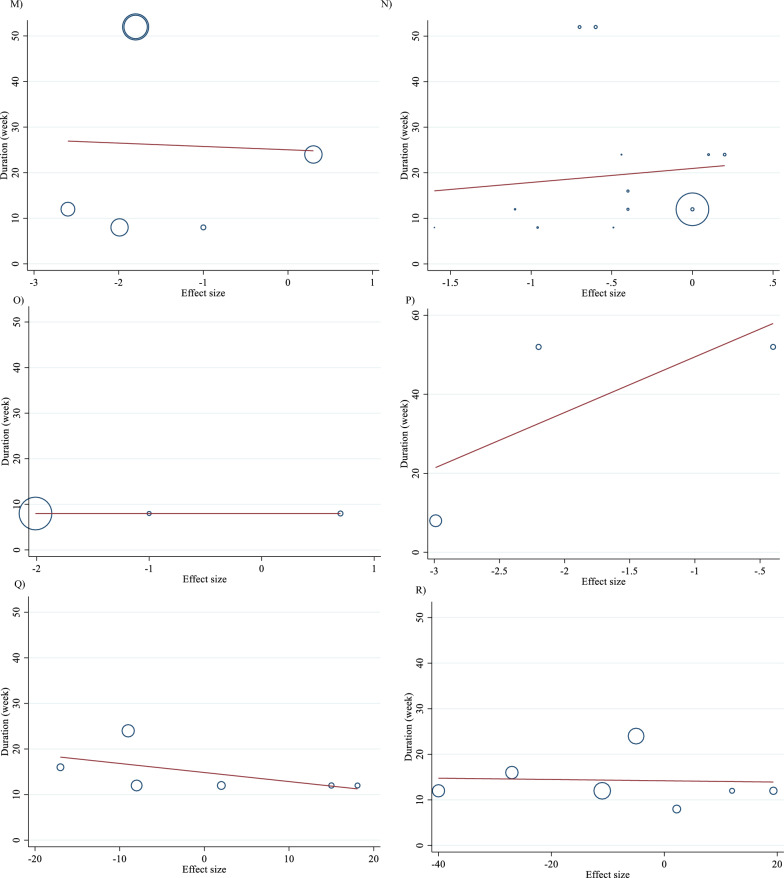
Linear dose–response relations between carnitine consumption and absolute mean differences. Dose–response relations between duration of intervention (week) and absolute mean differences in **A** TG (mg/dl); **B** TC (mg/dl); **C** LDL (mg/dl); **D** HDL (mg/dl); **E** FBG (mg/dl); **F** Insulin (uU/ml); **G** HbA1c (%); **H** HOMA-IR; **I** SBP (mmHg); **J** DBP (mmHg); **K** CRP (mg/l); **L** TNF-α (pg/ml); **M** weight (kg); **N** BMI (kg/m^2^); **O** BFP (%); P) Leptin (ng/ml); **Q** Apo A (mg/dl) and **R** Apo B (mg/dl). *Apo A* Apolipoprotein A, *Apo B* Apolipoprotein B; *BFP* body fat percentage, *BMI* body mass index, *CI* confidence interval, *CRP* c-reactive protein, *FBG* fasting blood glucose, *HbA1c* hemoglobin A1c, *HDL* high-density lipoprotein, *HOMA-IR* homeostatic model assessment for insulin resistance, *LDL* low-density lipoprotein, *DBP* diastolic blood pressure, *SBP* systolic blood pressure, *TC* total cholesterol, *TG* triglyceride, *TNF-α* tumor necrosis factor alpha, *WMD* weighted mean differences

Meta-regression analyses were performed to assess whether TG, TC, LDL, HDL, FBG, insulin, HbA1c, HOMA-IR, SBP, DBP, CRP, TNF-α, weight, BMI, BFP, Leptin, apo A and apo B were affected by L-carnitine doses and intervention durations. We did not find a significant linear relationship between dose (g/day) (coefficients = -0.01, p = 0.969) and duration (weeks) (coefficients = 0.01, p = 0.879) of intervention and changes in TG (Figs. 5A and 6A). Also, we did not find a significant linear relationship between dose (g/day) (coefficients = − 0.02, p = 0.497) and duration (weeks) (coefficients = 0.16, p = 0.427) of intervention and changes in TC (Figs. 5B and 6B). In addition, we did not find a significant linear relationship between dose (g/day) (coefficients = − 0.01, p = 0.982) and duration (weeks) (coefficients = − 0.03, p = 0.853) of intervention and changes in LDL (Figs. 5C and 6C). We did not find a significant linear relationship between dose (g/day) (coefficients = 0.01, p = 0.982) and duration (weeks) (coefficients = 0.48, p = 0.466) of intervention and changes in HDL (Figs. 5D and 6D). There was not a significant linear relationship between dose (g/day) (coefficients = − 0.01, p = 0.925) and duration (weeks) (coefficients = 0.48, p = 0.466) of intervention and changes in FBG (Figs. 5E and 6E). Also, there was not a significant linear relationship between dose (g/day) (coefficients = − 0.01, p = 0.982) and duration (weeks) (coefficients = − 0.28, p = 0.481) of intervention and changes in insulin (Figs. 5F and 6F). In addition, there was not a significant linear relationship between dose (g/day) (coefficients = − 0.08, p = 0.510) and duration (weeks) (coefficients = − 1.74, p = 0.537) of intervention and changes in HbA1c (Figs. 5G and 6G). Also, there was not a significant linear relationship between dose (g/day) (coefficients = − 0.30, p = 0.398) and duration (weeks) (coefficients = − 3.99, p = 0.513) of intervention and changes in HOMA-IR (Figs. 5H and 6H). We did not find a significant linear relationship between dose (g/day) (coefficients = -0.46, p = 0.474) and duration (weeks) (coefficients = 2.04, p = 0.630) of intervention and changes in SBP (Figs. 5I and 6I). Also, we did not find a significant linear relationship between dose (g/day) (coefficients = − 0.25, p = 0.593) and duration (weeks) (coefficients = − 0.81, p = 0.737) of intervention and changes in DBP (Figs. 5J and 6J). In addition, we did not find a significant linear relationship between dose (g/day) (coefficients = − 0.05, p = 0.929) and duration (weeks) (coefficients = 159.58, p = 0.182) of intervention and changes in CRP (Figs. 5K and 6K). We did not find a significant linear relationship between dose (g/day) (coefficients = 0, p = 1.000) and duration (weeks) (coefficients = 13.03, p = 0.105) of intervention and changes in TNF-α (Figs. 5L and 6L). There was not a significant linear relationship between dose (g/day) (coefficients = − 0.13, p = 0.835) and duration (weeks) (coefficients = − 0.73, p = 0.947) of intervention and changes in weight (Figs. 5M and 6M). There was not a significant linear relationship between dose (g/day) (coefficients = − 0.11, p = 0.857) and duration (weeks) (coefficients = 3.07, p = 0.731) of intervention and changes in BMI (Figs. 5N and 6N). There was not a significant linear relationship between dose (g/day) (coefficients = − 0.43, p = 0.644) and duration (weeks) (coefficients = 0, p = 1.000) of intervention and changes in BFP (Figs. 5O and 6O). There was not a significant linear relationship between dose (g/day) (coefficients = 0, p = 1.000) and duration (weeks) (coefficients = 14.07, p = 0.474) of intervention and changes in leptin (Figs. 5P and 6P). There was not a significant linear relationship between dose (g/day) (coefficients = 0.02, p = 0.836) and duration (weeks) (coefficients = − 0.19, p = 0.368) of intervention and changes in apo A (Figs. 5Q and 6Q). There was not a significant linear relationship between dose (g/day) (coefficients = 0.01, p = 0.899) and duration (weeks) (coefficients = − 0.01, p = 0.917) of intervention and changes in apo B (Figs. 5R and 6R).

### Publication bias

**Fig. 7:**
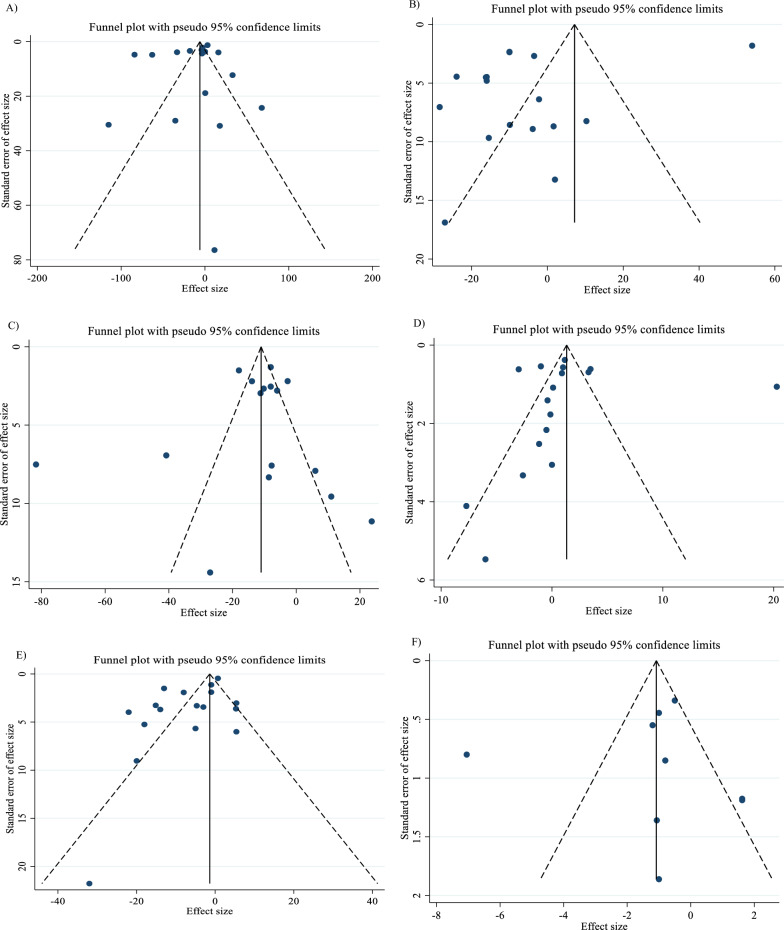

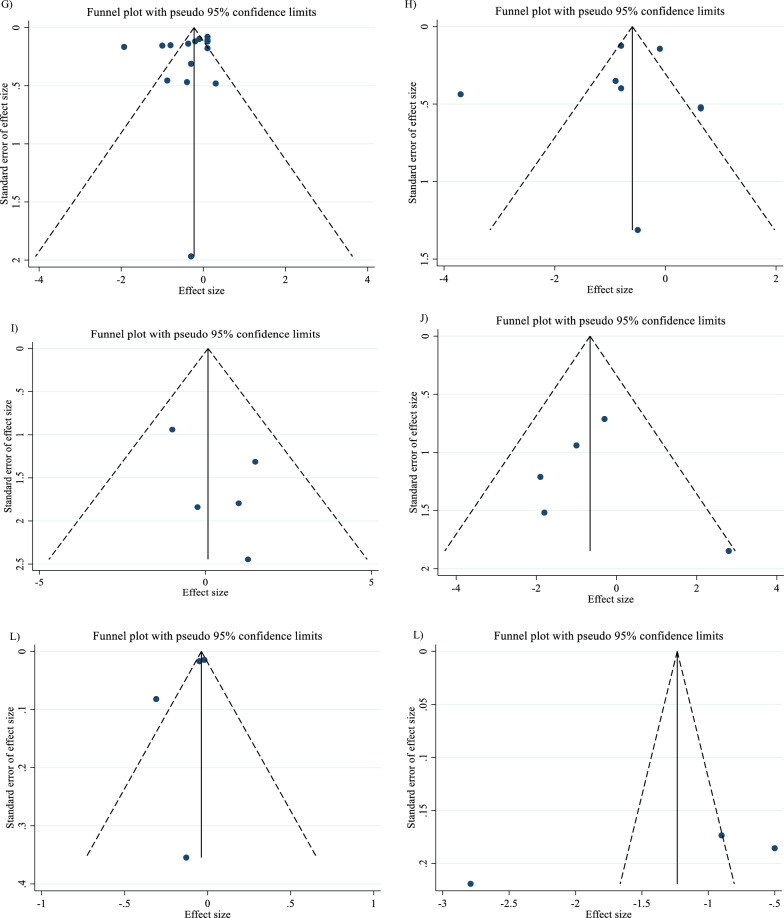

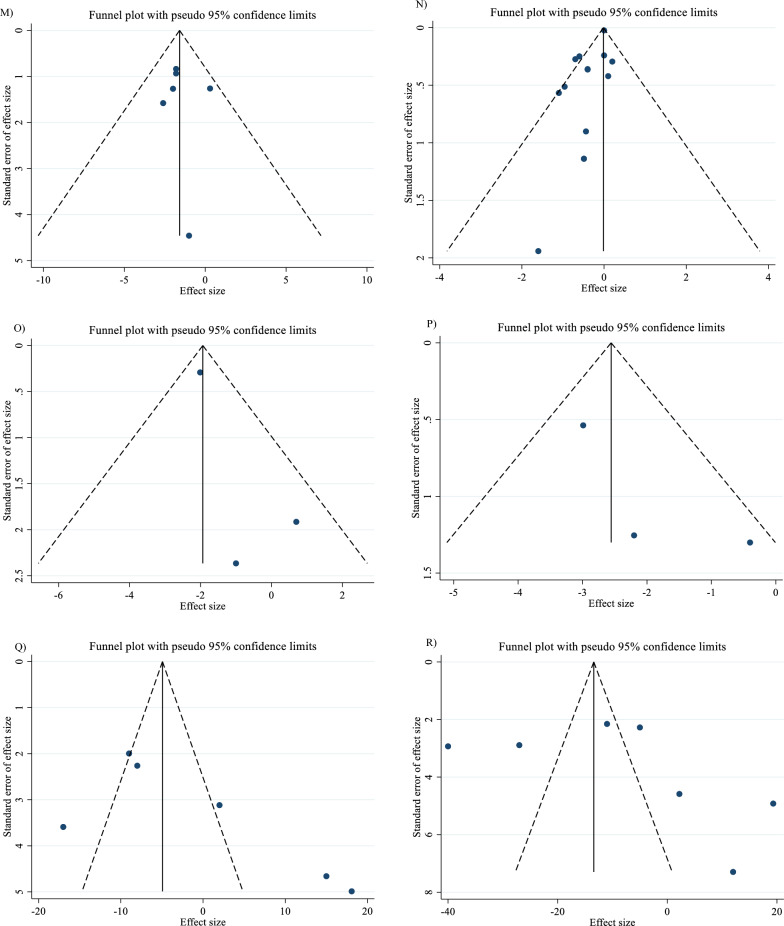
Funnel plots for the effect of carnitine consumption on **A** TG (mg/dl); **B** TC (mg/dl); **C** LDL (mg/dl); **D** HDL (mg/dl); **E** FBG (mg/dl); **F** Insulin (uU/ml); **G** HbA1c (%); H) HOMA-IR; I) SBP (mmHg); **J** DBP (mmHg); **K** CRP (mg/l); **L** TNF-α (pg/ml); **M** weight (kg); **N** BMI (kg/m^2^); **O** BFP (%); **P** Leptin (ng/ml); **Q** Apo A (mg/dl) and **R** Apo B (mg/dl). *Apo A* Apolipoprotein A, *Apo B* Apolipoprotein B, *BFP* body fat percentage, *BMI* body mass index, *CI* confidence interval, *CRP* c-reactive protein, *FBG* fasting blood glucose, *HbA1c* hemoglobin A1c, *HDL* high-density lipoprotein, *HOMA-IR* homeostatic model assessment for insulin resistance; *LDL* low-density lipoprotein, *DBP* diastolic blood pressure, *SBP* systolic blood pressure, *TC* total cholesterol, *TG* triglyceride, TNF-α tumor necrosis factor alpha, *WMD* weighted mean differences

Although the visual inspection of funnel plots showed slight asymmetries, no significant publication bias was detected for TG (P _Egger’s test_ = 0.281, P _Begg’s test_ = 0.303; Fig. 7A), LDL (P _Egger’s test_ = 0.710, P _Begg’s test_ = 0.893; Fig. 7C), HDL (P _Egger’s test_ = 0.927, P _Begg’s test_ = 0.174; Fig. 7D), insulin (P _Egger’s test_ = 0.859, P _Begg’s test_ = 1.000; Fig. 7F), HbA1c (P _Egger’s test_ = 0.274, P _Begg’s test_ = 0.113; Fig. 7G), HOMA-IR (P _Egger’s test_ = 0.763, P _Begg’s test_ = 0.711; Fig. 7H), SBP (P _Egger’s test_ = 0.271, P _Begg’s test_ = 1.000; Fig. 7I), DBP (P _Egger’s test_ = 0.818, P _Begg’s test_ = 1.000; Fig. 7J), CRP (P _Egger’s test_ = 0.320, P _Begg’s test_ = 0.734; Fig. 7K), TNF-α (P _Egger’s test_ = 0.307, P _Begg’s test_ = 1.000; Fig. 7L), weight (P _Egger’s test_ = 0.8331, P _Begg’s test_ = 1.000; Fig. 7M), BFP (P _Egger’s test_ = 0.330, P _Begg’s test_ = 1.000; Fig. 7O), Leptin (P _Egger’s test_ = 0.335, P _Begg’s test_ = 0.296; Fig. 7P), apo A (P _Egger’s test_ = 0.121, P _Begg’s test_ = 0.133; Fig. 7Q) and apo B (P _Egger’s test_ = 0.425, P _Begg’s test_ = 0.548; Fig. 7R). The funnel plot and statistical test showed no evidence of a publication bias for TG, LDL, HDL, insulin, HbA1c, HOMA-IR, SBP, DBP, CRP, TNF-α, weight, BFP, Leptin, apo A and apo B. However, Egger's test or Begg's test showed significant asymmetry for FBG (P _Egger’s test_ = 0.021, P _Begg’s test_ = 0.592; Fig. 7E), TC (P _Egger’s test_ = 0.089, P _Begg’s test_ = 0.029; Fig. 7B) and BMI (P _Egger’s test_ = 0.007, P _Begg’s test_ = 0.870; Fig. 7N).

### Sensitivity analysis

According to the sensitivity analysis, no study affected the overall results of LDL, HDL, FBG, HbA1c, SBP, DBP, weight, apo A, and apo B after removing individual study effects, although Y Liang et al. 1998 (WMD: − 10.52, CI 95% − 23.43, 2.38), SS Santo et al. 2006 (WMD: − 14.66, CI 95%: − 29.83, 0.50), M Malaguarnera et al. 2009 (WMD: − 8.00, CI 95% − 18.31, 2.31), F Galvano et al. 2009 (WMD: − 9.60, CI 95% − 21.66, 2.45), A Hassani &M Ghorbani. 2018 (WMD: − 14.20, CI 95% − 29.06, 0.65), YMH Bruls et al. 2019 (WMD: − 12.81, CI 95% − 25.84, 0.22) and HM El-Sheikh et al. 2019 (WMD: − 11.88, CI 95% − 25.15, 1.38) [32, 36–38, 40, 46, 47] affected the overall results of TG, A Hassani &M Ghorbani. 2018 (WMD: -10.99, CI 95% − 15.16, − 6.81) [38] affected the overall results of TC, HM El-Sheikh et al. 2019 (WMD: − 0.58, CI 95% − 1.14, − 0.01) [36] affected the overall results of insulin, A Molfino et al. 2010 (WMD: − 0.69, CI 95% − 1.70, 0.30), G Derosa et al. 2011 (WMD: − 0.69, CI 95% − 1.46, 0.08), G Derosa et al. 2011 (WMD: − 0.70, CI 95% − 1.47, 0.05), A Parvanova et al. 2018 (WMD: − 0.82, CI 95% − 1.71, 0.06), HM El-Sheikh et al. 2019 (WMD: − 0.33, CI 95% − 0.79, 0.11) [34–36, 42, 43] affected the overall results of HOMA-IR, G Derosa et al. 2011 (WMD: − 0.14, CI 95%: − 0.40, 0.10) and G Derosa et al. 2011 (WMD: − 0.16, CI 95%: − 0.38, 0.06) [34, 35] affected the overall results of CRP, TNF-α and BMI, RJ Bloomer et al. 2009 (WMD: − 1.31, CI 95% − 3.63, 1.00) and A Barzegar et al. 2013 (WMD: 0.02, CI 95% − 2.88, 2.94) [29, 31] affected the overall results of BFP, G Derosa et al. 2011 (WMD: − 1.96, CI 95%: − 4.44, 0.51) and A Barzegar et al. 2013 (WMD: − 1.33, CI 95%: − 3.10, 0.43) [29, 35] affected the overall results of leptin.

### GRADE assessment

The GRADE evidence profile and the certainty in outcomes of L-carnitine supplementation on TG, TC, LDL, HDL, FBG, insulin, HbA1c, HOMA-IR, SBP, DBP, CRP, TNF-α, weight, BMI, BFP, Leptin, apo A and apo B were shown in supplementary Table 3. The quality of evidence was very low due to the inconsistency for HbA1c, HOMA-IR, CRP, and TNF-α and imprecision for SBP and DBP. The quality of evidence was low due to the inconsistency and risk of bias for TG, LDL, and FBG and inconsistencies and imprecision for insulin. Although The quality of evidence was moderate due to the inconsistency, risk of bias, and imprecision for HDL, apo A, and apo B. The quality of evidence was high due to the inconsistency, risk of bias, imprecision, and publication bias for TC.

## Discussion

In this meta-analysis, the impact of L-carnitine on lipid profile, glycemic index, BP, inflammatory markers, and anthropometric measures was explored in patients with diabetes and glucose intolerance. The findings revealed that L-carnitine supplementation had a significant positive effect on reducing levels of TG, LDL, FBG, HbA1c, HOMA-IR, CRP, TNF-α, weight, BMI, BFP, and leptin. However, no significant effect was observed on TC, HDL, serum insulin, SBP, DBP, apo A and apo B in these patients. Meta-regression analysis did not indicate any significant relationship between the dosage and duration of L-carnitine supplementation and the variables measured. Non-linear dose–response analysis suggested that optimal improvement in FBG, HbA1c and HOMA-IR occurred after approximately 50 weeks of L-carnitine supplementation. It was also found that the duration of supplementation equal to or greater than 25 weeks had a decreasing effect on weight. The findings of this meta-analysis indicate that the consumption of L-carnitine leads to a decrease in FBG, HbA1c, and HOMA-IR levels by approximately 6.24 mg/dl, 0.37%, and 0.72 units respectively in individuals with diabetes and glucose intolerance. In a meta-analysis conducted by Fathizadeh et al. involving 37 RCTs, it was observed that L-carnitine supplementation has a significant impact on reducing insulin levels despite FPG, HOMA-IR, and HbA1c levels. [49]. In contrast to the findings of the meta-analysis presented, a separate study conducted in 2012 on four randomized controlled trials involving 284 patients with T2D demonstrated that L-carnitine supplementation resulted in a significant decrease in FBG levels, but did not have an impact on levels of HbA1c [50]. Additionally, the meta-analysis conducted by Xu et al. in 2017, which examined 5 RCTs, determined that the prolonged consumption of L-carnitine supplement, exceeding a duration of 9 months, has notable impacts on HOMA-IR levels among individuals with insulin resistance (IR) [51].

Based on the subgroup analysis conducted in this study, it was found that L-carnitine demonstrated greater effectiveness in reducing FBG and insulin levels when administered for a duration exceeding 12 weeks and at a dosage of more than 2 g/day to obese or overweight patients. Additionally, research has confirmed that obese individuals exhibit decreased concentrations of plasma L-carnitine [52]. It is possible that the consumption of a high dosage of L-carnitine can potentially compensate the deficiency of carnitine in individuals who are obese, leading to positive effects on glycemic control. Considering the results of this meta-analysis and other studies [53], L-carnitine supplementation seems to have positive effects on inflammation markers, and for this reason, it might help to improve the glycemic factors [54].

Based on the nonlinear dose–response analysis, it has been observed that the administration of L-carnitine can lead to a decrease in levels of FBG, HbA1c, and HOMA-IR starting from the 12th week. However, the most significant reduction in these markers is observed after approximately 50 weeks of continuous L-carnitine consumption. Since diabetic patients with other risk factors such as metabolic syndrome are more susceptible to CVD, controlling blood sugar and other CVD factors in these patients are very important [55, 56]. Some studies have shown that carnitine deficiency leads to impaired insulin sensitivity and high blood glucose [57]. Having said that, there have suggested several mechanisms by which L-carnitine would have beneficial effects on glucose metabolism [54]. The presence of high levels of long-chain Acyl-CoAs and other fatty acid metabolites in muscle and heart tissue is associated with a decline in insulin signaling and the development of insulin resistance (IR). However, the addition of carnitine promotes the mitochondrial oxidation of long-chain Acyl-CoAs, resulting in improved control over blood sugar levels [58, 59]. The supplementation of carnitine has been found to enhance the activity of genes associated with glucose transportation, such as GLUT8. Additionally, it has been observed to decrease the expression of Phosphoenolpyruvate Carboxykinase 1 (PCK1) and Fructose-bisphosphatase 2 (FBP2), both of which play a role in gluconeogenesis [60]. Carnitine has been found to have an impact on the insulin-like growth factor (IGF-1) signaling pathway and IGF-binding proteins, potentially contributing to the regulation of glucose levels [61]. Considering the previous studies, carnitine has been shown to have anti-inflammatory and antioxidant properties [62] along with decreasing body weight, and adipose tissue [63] and improving IR [64, 65]. When taken together, the combined effects of L-carnitine are believed to enhance insulin sensitivity and glucose metabolism. The findings of this meta-analysis indicate that supplementation with L-carnitine can lead to a significant reduction in TG levels by 13.50 mg/dl and LDL-C levels by 12.66 mg/dl. However, no significant impact was observed on TC, HDL-C, apo A, and apo B levels. Similarly, in a meta-analysis conducted by Asbaghi et al. in 2020, it was found that L-carnitine supplementation significantly decreased TC and LDL levels, but had no effect on HDL-C and TG levels [66]. An older meta-analysis in 2013 included 4 RCTs with 284 T2D patients. In this study, results showed a considerable lowering effect of carnitine on TC, LDL, apo A, and apo B, unlike TG levels [50]. The variation in the findings may be attributed to the inclusion of a larger number of studies and a greater sample size in our analysis. The findings of a meta-analysis conducted in 2019, which examined 67 randomized controlled trials, provided a comprehensive overview of the impact of L-carnitine consumption on blood lipid levels in both patients and healthy individuals. The results indicated a significant decrease in TG, TC, and LDL levels, as well as an increase in HDL levels [67]. In an academic context, it was found through a meta-analysis that L-carnitine supplementation has beneficial effects on TC and TG levels in overweight patients with liver disease. This is observed when doses are below 2 g/d and the supplementation lasts for more than 24 weeks. [68]. Also in line with our findings, another study did not find a significant effect of carnitine on apo A and apo B [69] but in another one, a significant reduction was seen [50]. It is worth noting that this meta-analysis included only 4 RCTs and only two of them investigated apo A and apo B as outcomes. The main apo-lipoprotein for HDL is apo A [50] and since no significant change was observed in its level in the present study, we did not expect any considerable change in HDL-C level as well.

In the current study, subgroup analysis demonstrated that there was a significant reduction in TC levels under certain conditions. These conditions included a baseline level of over 200 mg/dl, a trial duration of more than 12 weeks, an intake dose of carnitine exceeding 2 g/day, and a BMI exceeding 30. Additionally, L-carnitine exhibited significant lowering effects on TG and LDL-C levels under specific circumstances. These circumstances consisted of a trial duration of more than 12 weeks and serum levels of TG over 150 mg/dl and LDL-C over 100 mg/dl. Furthermore, it was observed that higher tissue concentrations of L-carnitine were associated with greater improvements in lipid metabolism [70, 71] and this may explain why it is more effective at high and long-term doses.

Dyslipidemia is common among diabetic patients and those with glycemic abnormalities [72]. As suggested by the National Cholesterol Education Program (NCEP), patients with diabetes should improve the risk factors to reduce the overall risk of CVD [73]. Since years ago studies have reported the positive effects of L-carnitine on lipid profile in diabetic patients [30]. L-carnitine supplementation can have beneficial impacts on lipid profile through several mechanisms. It has been shown that L-carnitine reduces fatty acids conversion to triglycerides by its role in beta-oxidation of fatty acids in muscle cells and hepatocytes [74, 75] along with reducing the accumulation of short and medium-chain fatty acids in mitochondria [76]. Studies have shown that IR and TG are directly related to each other [77]. As shown in the present meta-analysis and other ones, L-carnitine supplementation can reduce IR and HOMA-IR amounts [69]. Thus, decreasing IR can lead to reducing TG levels.

Also, our meta-analysis did not show any notable effect of carnitine on SBP and DBP even in subgroup analyses. Choi et al. concluded from their results that L-carnitine had significantly lowered SBP unlike DBP [78] in addition to another study that showed an effective decrease in SBP in nondiabetic hypertensive patients who took 2 g/d of carnitine [79]. Another one examined 10 RCTs in 2019 and concluded that L-carnitine supplementation has a lowering effect on DBP without affecting SBP levels [80]. The results of these variables are contradictory and it seems further studies are needed for a comprehensive conclusion.

L-carnitine has been shown to have beneficial effects on inflammation and dyslipidemia [81, 82]. Improving these main risk factors can ameliorate blood pressure [83]. In addition, L-carnitine supplementation increases TMAO in the blood which has a role in decreasing blood pressure [84].

This systematic review and meta-analysis showed that L-carnitine supplementation has a significant lowering effect on CRP, TNF-α, and leptin levels by 0.07 mg/dl, 1.39 pg/ml, and 2.21 ng/ml, respectively. To date, there has been a lack of meta-analyses examining the impact of L-carnitine on inflammatory markers in individuals specifically diagnosed with diabetes or glucose intolerance. This current study serves as the inaugural investigation in this area. A previous meta-analysis conducted by Haghighatdoost et al. in 2018 examined 13 randomized controlled trials (RCTs) and found a notable decrease in inflammatory mediators following L-carnitine intervention lasting longer than 12 weeks in adult participants [85]. Consistent with the findings of our research, a separate meta-analysis indicated that the supplementation of L-carnitine resulted in a decrease in CRP and TNF-α levels among both healthy individuals and those with specific disorders [86]. Additionally, the findings of our study align with a meta-analysis that consisted of 7 randomized controlled trials (RCTs). This meta-analysis demonstrated that the consumption of L-carnitine at doses exceeding 3 g/day for a duration of less than 12 weeks exhibits a significant impact on leptin levels in individuals with diabetes [87].

The analysis of subgroups based on CRP levels indicated that the administration of L-carnitine demonstrated positive outcomes in both interventions, regardless of the duration being less than or more than 12 weeks. Studies have shown that an increase in inflammatory factors can lead to atherosclerosis and CVD, and anti-inflammatory interventions may improve this issue [88, 89]. L-carnitine can ameliorate inflammation factor levels through different mechanisms. Reactive Oxygen Species (ROS) are well known to be enhancers of inflammation [90]. L-carnitine can reduce ROS production and therefore decrease inflammatory response [91]. In addition, carnitine can downregulate the nuclear factor kappa B (NF-κB) pathway and in the following suppress the expression of pro-inflammatory cytokines [92]. Moreover, L-carnitine can upregulate peroxisome proliferator-activated receptor γ (PPAR γ) which plays an important role in the regulation of oxidative stress [93, 94]. Concerning Leptin, as its level is proportional to the body fat mass, the significant decrease in the present study could be due to the reduction of BFP [95]. Besides all of these, since carnitine can decrease body weight and body fat mass [96], TNF-α levels can be decreased as well since it is secreted by fat tissue cells [97].

This meta-analysis showed that consuming L-carnitine can significantly lower BMI, weight, and BFP by 0.28 kg/m^2^, 1.58 kg, and 1.83%, respectively. However, subgroup analysis showed this impact was more effective in obese patients. Furthermore, the non-linear dose–response showed an association between L-carnitine supplementation and body weight, indicating a decreasing trend of body weight in durations more than 25 weeks. A meta-analysis of 10 RCT studies done by Wang in 2021 showed that 2 g/day of L-carnitine for at least 2 weeks is required to lower BMI in T2D patients [98]. Another meta-analysis done in 2019 consisting of 37 RCTs, revealed that L-carnitine supplementation significantly decreases body weight, BMI, and fat mass without any effect on BFP. It also mentioned that 2 g/d ingestion of l-carnitine has the maximum effect on weight in adults [96].

Subgroup analysis on weight shows that L-carnitine supplementation is more effective in durations more than 12 weeks and taken by obese individuals. It might be because of this fact that obese individuals have low levels of carnitine [52] and longer interventions might help them to provide carnitine reserves, thus showing better results. According to the nonlinear dose–response analysis, L-carnitine supplementation causes weight loss in intervention durations of more than 24 weeks but the optimum duration of it for effective weight loss is near 50. The precise mechanism through which L-carnitine supplementation affects weight and body composition has not been elucidated in detail. However, it is possible that these effects can be attributed to the involvement of carnitine acyltransferases (CATs), enzymes that play a crucial role in energy balance and fat metabolism. [99, 100]. Also, L-carnitine has the potential to elevate acetyl-coenzyme A levels, which is a byproduct of beta-oxidation. Consequently, this can impact the brain's glucose supply, thereby playing a role in modulating energy expenditure and regulating appetite control [101, 102].

It is important to note that this systematic review and meta-analysis were conducted with several strengths. Firstly, the study is comprehensive as it analyzes the effects of L-carnitine supplementation on all related risk factors of cardiovascular disease in patients with diabetes and glucose intolerance. Secondly, all eligible RCTs were included in the analysis. Thirdly, various types of analyses, such as subgroup analysis, sensitivity analysis, GRADE assessment, dose–response nonlinear analysis, and publication bias assessment, were performed. Fourthly, the overall sample size of the included studies is substantial. Fifthly, there were no restrictions on language or time during the search for relevant studies. Sixthly, adverse effects reported in the trials were stratified by gender. Seventhly, due to the inclusion of studies from different countries, the findings can be generalized to a larger population. However, it is important to acknowledge that there may be some limitations that need to be considered. In the academic context, there are several factors that can affect the validity and reliability of research studies. Firstly, poor participant follow-up and non-compliance can undermine the study's findings. Additionally, some studies fail to report the reasons for participant withdrawal. Secondly, certain studies do not consider or assess important factors such as participants' diets, physical activity levels, smoking habits, and other potential variables that could influence results. Inadequate adjustment for confounding variables is also observed in some studies. Thirdly, variations in L-carnitine doses and intake durations among different studies can lead to inconsistent findings. Furthermore, the use of different brands of carnitine with varying bioavailability can introduce further variation in results. Fourthly, discrepancies arise from the use of different biochemical measurement kits and methods in laboratories as well as differences in the accuracy of weight measurement tools. Fifthly, a high degree of heterogeneity exists in the findings across studies within this field. Sixthly, comprehensive analyses that consider all relevant variables are lacking in some studies. Randomization procedures may also be improperly implemented, leading to publication bias. Seventhly, certain studies lack baseline control measures. Eighthly, adverse side effects resulting from interventions are not consistently reported among participants. Lastly, some studies fail to report primary outcomes.

## Conclusion

Based on our analysis of 21 RCTs, we found that supplementing with L-carnitine can lead to significant improvements in various health markers, such as TG, LDL, FBG, HbA1c, HOMA-IR, CRP, TNF-α, weight, BMI, BFP, and leptin levels in patients with diabetes and glucose intolerance. However, there was no significant effect observed on TC, HDL, serum insulin, SBP, DBP, apo A and apo B levels. Our analysis also revealed that the optimal duration for L-carnitine supplementation to effectively reduce FBG, HbA1c, and HOMA-IR was approximately 50 weeks after initiation. The findings suggest that longer durations of supplementation (≥ 25 weeks) have a diminishing impact on weight. Due to the significant risk of bias in the majority of trials included, additional well-designed and comprehensive RCTs with larger sample sizes and robust analytical approaches are necessary to ascertain the influence of L-carnitine on CVD risk factors in individuals with diabetes and glucose intolerance.

### Supplementary Information

Supplementary Material 1.

Supplementary Material 2.

Supplementary Material 3.

## Data Availability

No datasets were generated or analysed during the current study.

## Abbreviations

RCTs: Randomized clinical trials
WMD: Weighted mean differences
CI: Confidence intervals
BMI: Body mass index
GRADE: Grading of Recommendations Assessment, Development, and Evaluation
PRISMA: Preferred reporting items for systematic reviews and meta-analyses
PICO: Participant, intervention, comparison/control, and outcome
CVDs: Cardiovascular diseases
LDL-C: Low-density lipoprotein cholesterol
HDL-C: High-density lipoprotein cholesterol
TC: Total cholesterol
TG: Triglycerides
Apo A: Apolipoprotein A
Apo B: Apolipoprotein B
SD: Standard deviation
SEs: Standard errors
IQRs: Interquartile ranges
TMAO: Trimethylamine-N-oxide
HMG-CoA: B-hydroxy b-methylglutaryl co-A
SEs: Standard errors
IR: Insulin resistance
PDHC: Pyruvate dehydrogenase complex
GLUT8: Glucose transporter 8
PCK1: Phosphoenolpyruvate carboxykinase 1
FBP2: Fructose-bisphosphatase 2
IGF-1: Insulin-like growth factor
NAFLD: Nonalcoholic fatty liver disease
NCEP: National Cholesterol Education Program
HMG-CoA: β-Hydroxy β-methyl-glutaryl coenzyme A
NF-κB: Nuclear factor kappa B
PPARγ: Peroxisome proliferator-activated receptor γ
CATs: Carnitine acyltransferases
SBP: Systolic blood pressure
COT: Carnitine octanoyl transferase
TNF- α: Tumor necrosis factor-alpha
DBP: Diastolic blood pressure
CRP: , C-reactive protein
IL-6: Interleukin-6
WC: Waist circumference
Apo A: Apolipoprotein A
Apo B: Apolipoprotein B
BFP: Body fat percentage
HOMA-IR: Homeostasis model assessment-insulin resistance
HbA1c: Hemoglobin A1c
FBG: Fasting blood glucose
ROS: Reactive oxygen species
