# Supplementary material for: The effects of L-carnitine supplementation on cardiovascular risk factors in participants with impaired glucose tolerance and diabetes: a systematic review and dose–response meta-analysis
Source: Diabetol Metab Syndr. 2024 Jul 31;16:185. doi: 10.1186/s13098-024-01415-8 (PMC11290177; doi:10.1186/s13098-024-01415-8)
Supplement: Supplementary file 1 — Supplementary Material 1. [file 13098_2024_1415_MOESM1_ESM.docx]

**supplementary Table 1.** Characteristics of included studies in the meta-analysis.

| Studies | Country | Study Design | Participant | Sample size and Sex | Sample size | | Trial Duration  (Week) | Means Age | | Means BMI | | Intervention | | Adverse events |
| --- | --- | --- | --- | --- | --- | --- | --- | --- | --- | --- | --- | --- | --- | --- |
|  |  |  |  |  | **IG** | **CG** |  | **IG** | **CG** | **IG** | **CG** | **carnitine (g/d)** | **Control group** |  |
| Y Liang et al. 1998 | China | Parallel, R, PC, DB | Diabetic Patients | M/F (F:30, M:16) | 23 | 23 | 12 | 59.4±1.7 | 57.9±2.6 | 27.2±3.1 | 26.9±2.8 | 3 | Placebo | NR |
| G Derosa et al. 2003 | Italy | Paralell, R, PC, DB | Type 2 Diabetes Mellitus | M/F (F:47, M:47) | 46 | 48 | 24 | 52±6 | 50±7 | 27.3±2.5 | 26.8±2.2 | 2 | Placebo | No adverse events |
| AR Rahbar et al. 2005 | Iran | Paralell, R, PC, DB | Type 2 Diabetes Mellitus | M/F (F:13, M:22) | 19 | 16 | 12 | 50.5±4.8 | 52.2±2.6 | 27.9±2 | 28.2±1.52 | 3 | Placebo | No adverse events |
| V Solfrizzi et al. 2006 | Italy | Parallel, R, PC | Type 2 Diabetes Mellitus | M/F (F:25, M:27) | 26 | 26 | 8 | 65.4±10.4 | 62.7±8.74 | 29±4.5 | 27.8±4.4 | 2 | control diet | No adverse events |
| SS Santo et al. 2006 | Italy | Parallel, R, PC, DB | Type 2 Diabetes Mellitus | M/F: 74 | 37 | 37 | 12 | 61.75±3.03 | 61.26±1.6 | 34±0.02 | 36±0.01 | 2 | Placebo | NR |
| M González-Ortiz et al. 2008 | Mexico | Paralell, R, PC, DB | Type 2 Diabetes Mellitus | M/F (F:6, M:6) | 6 | 6 | 4 | 44.1±7.3 | 42.6±9.2 | 27.2±2.7 | 27.8±2.7 | 3 | Placebo | No adverse events |
| M Malaguarnera et al. 2009 | Italy | Paralell, R, PC | Type 2 Diabetes Mellitus | M/F (F:25, M:55) | 40 | 40 | 12 | 47±13 | 45±12 | 26.8±1.3 | 26.5±1.7 | 2 | control diet | NR |
| F Galvano et al. 2009 | Italy | Parallel, R, PC, DB | Type 2 Diabetes Mellitus | M/F (F:45, M:30) | 38 | 37 | 16 | 52.1±8.1 | 51.4±7.6 | 27.8±2 | 27.1±2.4 | 2 | control diet | No adverse events |
| M Malaguarnera et al. 2009 | Italy | Parallel, R, PC | Diabetic Patients | M/F (F:23, M:58) | 41 | 40 | 12 | 49±13 | 48±11 | 27.5±1.8 | 27.4±1.8 | 2 | placebo | gastrointestinal  tract complaints |
| RJ Bloomer et al. 2009 | USA | Parallel, R, PC, DB | pre-diabetics | M/F: 29 | 14 | 15 | 8 | 31±12 | 35±12 | 28.5±7.6 | 31.7±8.4 | 3 | Placebo | No adverse events |
| A Molfino et al. 2010 | Italy | Parallel, R, PC | Patients With Impaired Glucose Metabolism | M/F (F: 4, M:12) | 8 | 8 | 2 | 69.1±12.6 | 64.2±14.5 | 28.6±6.8 | 25.8±6.8 | 4 | placebo | No adverse events |
| G Derosa et al. 2011 | Italy | Parallel, R, PC, DB | Obese diabetic patients | M/F (F:131, M:127) | 132 | 126 | 52 | 51±4 | 53±6 | 32.9±2.8 | 33.1±2.9 | 2 | control diet | Flatulence, Constipation, Abdominal pain, Fatty/oily evacuation, Increased defecation, Fecal urgency, Malaise |
| G Derosa et al. 2011 | Italy | Parallel, R, PC, DB | Diabetic Patients | M/F (F:126, M:128) | 129 | 125 | 52 | 54±5 | 51±4 | 33.9±3.5 | 33.4±3.2 | 2 | control diet | No adverse events |
| A Barzegar et al. 2013 | Iran | Paralell, R, PC | Obese Type II Diabetes Mellitus | F: 60 | 30 | 30 | 8 | 37.03±6.1 | 36.7±5.6 | 33.4±2.78 | 33.7±2.81 | 2 | control diet | NR |
| MR Ramazanpour et al. 2015 | Iran | Parallel, R, PC | Diabetic patients | M: 20 | 10 | 10 | 4 | 51.6±2.98 | 50.8±2.2 | 24.7±0.6 | 25.5±0.59 | 0.5 | control diet | NR |
| M Ghorbani et al. 2017 | Iran | Paralell, R, PC, DB | Type 2 Diabetic Women | F: 20 | 10 | 10 | 8 | 52.7±1.6 | 52.7±1.6 | 29.82±4.35 | 29.82±4.35 | 0.5 | placebo | NR |
| A Hassani&M Ghorbani. 2018 | Iran | Paralell, R, PC, DB | Type 2 Diabetic Women | F: 20 | 10 | 10 | 8 | 52.2±6.8 | 53.6±3.2 | 29.36±4.34 | 28.41±4.06 | 0.5 | Placebo | NR |
| A Parvanova et al. 2018 | Italy | Parallel, R, PC, DB | Type 2 Diabetes Mellitus | M/F (F:64, M:165) | 116 | 113 | 24 | 64.9±7.7 | 64.6±7.5 | 30±4.7 | 30±5 | 2 | placebo | No adverse events |
| YMH Bruls et al. 2019 | Netherlands | Crossover, R, PC, DB | Volunteers with impaired glucose tolerance | M/F (F:3, M:20) | 11 | 12 | 4 | 62±6.8 | 61±6.9 | 29.7±1.6 | 28.9±2.1 | 2 | Placebo | NR |
| HM El-Sheikh et al. 2019 | Egypt | Parallel, R, PC | Type 2 Diabetes Mellitus | M/F (F:39, M:19) | 31 | 27 | 24 | 50.9±8.6 | 50.3±8.8 | 34.46±5.3 | 34.25±5.6 | 2 | control diet | No adverse events |
| N Talenezhad et al. 2020 | Iran | Paralell, R, PC, DB | Type 2 Diabetes Mellitus | M/F (F:43, M:27) | 35 | 35 | 12 | 56.4±8.7 | 54.9±7.95 | 28.63±3.6 | 29.54±3.8 | 1 | placebo | NR |

Abbreviations: IG, intervention group; CG, control group; DB, double-blinded; SB, single-blinded; PC, placebo-controlled; CO, controlled; R, randomized; NR, not reported; F, Female; M, Male.
