# Supplementary material for: The effects of L-carnitine supplementation on cardiovascular risk factors in participants with impaired glucose tolerance and diabetes: a systematic review and dose–response meta-analysis
Source: Diabetol Metab Syndr. 2024 Jul 31;16:185. doi: 10.1186/s13098-024-01415-8 (PMC11290177; doi:10.1186/s13098-024-01415-8)
Supplement: Supplementary file 2 — Supplementary Material 2. [file 13098_2024_1415_MOESM2_ESM.docx]

**Supplementary Table 2**. Subgroup analyses of L-carnitine on CVD risk factors in adults.

|  | **NO** | **WMD (95%CI)** | **P-value** | **Heterogeneity** | | |  |
| --- | --- | --- | --- | --- | --- | --- | --- |
|  |  |  |  | **P heterogeneity** | **I^2^** | **P between sub-groups** |  |
| Subgroup analyses of carnitine on serum TG (mg/dl) | | | | | | | |
| Overall effect | 17 | -13.50 (-26.33, -0.67) | **0.039** | <0.001 | 97.3% |  |  |
| Baseline TG (mg/dl) |  |  |  |  |  |  |  |
| <150 | 5 | -0.89 (-13.53, 11.75) | 0.890 | <0.001 | 90.9% | 0.100 |  |
| ≥150 | 12 | -19.94 (-38.79, -1.10) | **0.038** | <0.001 | 98.0% |  |  |
| Trial duration (week) |  |  |  |  |  |  |  |
| ˂12 | 6 | 5.49 (-13.39, 24.37) | 0.569 | 0.073 | 50.4% | **0.049** |  |
| ≥12 | 11 | -19.83 (-36.52, -3.15) | **0.020** | <0.001 | 98.3% |  |  |
| Intervention dose (g/day) |  |  |  |  |  |  |  |
| ˂2 | 2 | -2.95 (-7.22, 1.31) | 0.175 | 0.862 | 0.0% | 137 |  |
| ≥2 | 15 | -15.03 (-30.35, 0.28) | 0.054 | <0.001 | 97.6% |  |  |
| Baseline BMI (kg/m^2^) |  |  |  |  |  |  |  |
| Overweight (25-29.9) | 11 | -15.91 (-41.51, 9.71) | 0.224 | <0.001 | 97.8% | 0.779 |  |
| Obese (>30) | 5 | -10.25 (-22.66, 2.15) | 0.105 | <0.001 | 96.1% |  |  |
| Subgroup analyses of carnitine on serum TC (mg/dl) | | | | | | | |
| Overall effect | 17 | -6.49 (-21.93, 8.93) | 0.409 | <0.001 | 98.3% |  |  |
| Baseline TC (mg/dl) |  |  |  |  |  |  |  |
| <200 | 6 | 3.82 (-28.28, 35.93) | 0.815 | <0.001 | 98.7% | 0.314 |  |
| ≥200 | 11 | -12.82 (-17.45, -8.19) | **<0.001** | 0.003 | 63.0% |  |  |
| Trial duration (week) |  |  |  |  |  |  |  |
| ˂12 | 6 | 5.31 (-26.74, 37.37) | 0.745 | <0.001 | 96.5% | 0.280 |  |
| ≥12 | 11 | -12.52 (-16.95, -8.10) | **<0.001** | 0.001 | 67.3% |  |  |
| Intervention dose (g/day) |  |  |  |  |  |  |  |
| ˂2 | 2 | 22.59 (-40.01, 85.21) | 0.479 | <0.001 | 98.1% | 0.294 |  |
| ≥2 | 15 | -11.02 (-15.37, -6.68) | **<0.001** | <0.001 | 64.6% |  |  |
| Baseline BMI (kg/m^2^) |  |  |  |  |  |  |  |
| Overweight (25-29.9) | 11 | -4.33 (-29.62, 20.95) | 0.737 | <0.001 | 98.5% | 0.926 |  |
| Obese (>30) | 5 | -9.38 (-14.82, -3.94) | **0.001** | 0.010 | 69.7% |  |  |
| Subgroup analyses of carnitine on serum LDL (mg/dl) | | | | | | | |
| Overall effect | 16 | -12.66 (-18.12, -7.21) | **<0.001** | <0.001 | 91.5% |  |  |
| Baseline LDL (mg/dl) |  |  |  |  |  |  |  |
| <100 | 2 | 8.24 (-17.25, 33.74) | 0.526 | 0.020 | 81.5% | 0.081 |  |
| ≥100 | 14 | -15.03 (-20.78, -9.28) | **<0.001** | <0.001 | 91.3% |  |  |
| Trial duration (week) |  |  |  |  |  |  |  |
| ˂12 | 6 | -4.86 (-12.26, 2.53) | 0.198 | 0.140 | 39.8% | **0.027** |  |
| ≥12 | 10 | -16.17 (-22.97, -9.36) | **<0.001** | <0.001 | 94.5% |  |  |
| Intervention dose (g/day) |  |  |  |  |  |  |  |
| ˂2 | 2 | -8.05 (-12.81, -3.28) | **0.001** | 0.945 | 0.0% | 0.181 |  |
| ≥2 | 14 | -13.34 (-19.45, -7.23) | **<0.001** | <0.001 | 92.5% |  |  |
| Baseline BMI (kg/m^2^) |  |  |  |  |  |  |  |
| Overweight (25-29.9) | 10 | -7.23 (-11.59, -2.86) | **0.001** | 0.003 | 64.1% | **0.011** |  |
| Obese (>30) | 5 | -27.17 (-39.38, -14.96) | **<0.001** | <0.001 | 97.3% |  |  |
| Subgroup analyses of carnitine on serum HDL (mg/dl) | | | | | | | |
| Overall effect | 17 | 1.07 (-1.13, 3.28) | 0.341 | <0.001 | 96.2% |  |  |
| Baseline HDL (mg/dl) |  |  |  |  |  |  |  |
| <40 | 3 | 4.32 (-12.63, 21.29) | 0.617 | <0.001 | 98.8% | 0.648 |  |
| ≥40 | 14 | 0.37 (-0.85, 1.59) | 0.554 | <0.001 | 84.8% |  |  |
| Trial duration (week) |  |  |  |  |  |  |  |
| ˂12 | 6 | -0.81 (-2.58, 0.94) | 0.363 | 0.543 | 0.0% | 0.065 |  |
| ≥12 | 11 | 2.21 (-0.47, 4.90) | 0.107 | <0.001 | 97.6% |  |  |
| Intervention dose (g/day) |  |  |  |  |  |  |  |
| ˂2 | 2 | -0.29 (-2.46, 1.86) | 0.787 | 0.909 | 0.0% | 0.349 |  |
| ≥2 | 15 | 1.24 (-1.15, 3.65) | 0.309 | <0.001 | 96.6% |  |  |
| Baseline BMI (kg/m^2^) |  |  |  |  |  |  |  |
| Overweight (25-29.9) | 11 | -0.05 (-1.94, 1.83) | 0.954 | <0.001 | 87.2% | 0.358 |  |
| Obese (>30) | 5 | 4.21 (-1.62, 10.05) | 0.157 | <0.001 | 98.8% |  |  |
| Subgroup analyses of carnitine on serum FBG (mg/dl) | | | | | | | |
| Overall effect | 17 | -6.24 (-9.80, -2.69) | **0.001** | <0.001 | 91.0% |  |  |
| Trial duration (week) |  |  |  |  |  |  |  |
| ˂12 | 6 | 0.04 (-2.89, 2.99) | 0.974 | 0.170 | 35.5% | **0.003** |  |
| ≥12 | 11 | -9.44 (-14.92, -3.96) | **0.001** | <0.001 | 94.0% |  |  |
| Intervention dose (g/day) |  |  |  |  |  |  |  |
| ˂2 | 3 | -1.06 (-2.98, 0.86) | 0.279 | 0.364 | 1.0% | **0.020** |  |
| ≥2 | 14 | -7.14 (-11.86, -2.41) | **0.003** | <0.001 | 92.6% |  |  |
| Baseline BMI (kg/m^2^) |  |  |  |  |  |  |  |
| Overweight (25-29.9) | 11 | -6.29 (-10.96, -1.62) | **0.008** | <0.001 | 84.0% | 0.495 |  |
| Obese (>30) | 5 | -5.81 (-13.26, 1.63) | 0.128 | <0.001 | 96.2% |  |  |
| Subgroup analyses of carnitine on serum Insulin (uU/ml) | | | | | | | |
| Overall effect | 9 | -1.131 (-2.52, 0.26) | 0.112 | <0.001 | 88.4% |  |  |
| Trial duration (week) |  |  |  |  |  |  |  |
| ˂12 | 4 | 0.58 (-0.88, 2.05) | 0.436 | 0.292 | 19.5% | **0.027** |  |
| ≥12 | 5 | -2.03 (-3.83, -0.24) | **0.026** | <0.001 | 93.1% |  |  |
| Intervention dose (g/day) |  |  |  |  |  |  |  |
| ˂2 | 2 | 1.62 (-0.01, 3.25) | 0.053 | 1.000 | 0.0% | **0.003** |  |
| ≥2 | 7 | -1.82 (-3.35, -0.30) | **0.019** | <0.001 | 89.7% |  |  |
| Baseline BMI (kg/m^2^) |  |  |  |  |  |  |  |
| Overweight (25-29.9) | 5 | 0.13 (-1.10, 1.38) | 0.828 | 0.239 | 27.5% | **0.049** |  |
| Obese (>30) | 4 | -2.33 (-4.45, -0.20) | **0.031** | <0.001 | 94.8% |  |  |
| Subgroup analyses of carnitine on serum HbA1c (%) | | | | | | | |
| Overall effect | 15 | -0.37 (-0.67, -0.07) | **0.013** | <0.001 | 92.6% |  |  |
| Trial duration (week) |  |  |  |  |  |  |  |
| ˂12 | 4 | 0.09 (-0.06, 0.24) | 0.237 | 0.720 | 0.0% | **0.005** |  |
| ≥12 | 11 | -0.46 (-0.81, -0.11) | **0.010** | <0.001 | 93.9% |  |  |
| Baseline BMI (kg/m^2^) |  |  |  |  |  |  |  |
| Overweight (25-29.9) | 10 | -0.10 (-0.26, 0.04) | 0.157 | 0.042 | 48.4% | 0.123 |  |
| Obese (>30) | 5 | -0.70 (-1.44, 0.03) | 0.062 | <0.001 | 96.9% |  |  |
| Subgroup analyses of carnitine on HOMA-IR | | | | | | | |
| Overall effect | 8 | -0.72 (-1.40, -0.04) | **0.038** | <0.001 | 91.0% |  |  |
| Trial duration (week) |  |  |  |  |  |  |  |
| ˂12 | 4 | -0.01 (-1.01, 1.01) | 0.999 | 0.004 | 77.9% | 0.127 |  |
| ≥12 | 4 | -1.34 (-2.73, 0.05) | 0.059 | <0.001 | 95.3% |  |  |
| Intervention dose (g/day) |  |  |  |  |  |  |  |
| ˂2 | 2 | 0.64 (-0.08, 1.36) | 0.084 | 1.000 | 0.0% | **0.001** |  |
| ≥2 | 6 | -1.14 (-1.90, -0.37) | **0.004** | <0.001 | 92.4% |  |  |
| Baseline BMI (kg/m^2^) |  |  |  |  |  |  |  |
| Overweight (25-29.9) | 4 | -0.01 (-1.01, 1.01) | 0.999 | 0.004 | 77.9% | 0.127 |  |
| Obese (>30) | 4 | -1.34 (-2.73, 0.05) | 0.059 | <0.001 | 95.3% |  |  |
| Subgroup analyses of carnitine on SBP (mmHg) | | | | | | | |
| Overall effect | 5 | 0.07 (-1.16, 1.32) | 0.904 | 0.554 | 0.0% |  |  |
| Baseline SBP (mmHg) |  |  |  |  |  |  |  |
| <130 | 3 | -0.62 (-2.17, 0.93) | 0.434 | 0.667 | 0.0% | 0.142 |  |
| ≥130 | 2 | 1.32 (-0.75, 3.40) | 0.211 | 0.822 | 0.0% |  |  |
| Baseline BMI (kg/m^2^) |  |  |  |  |  |  |  |
| Overweight (25-29.9) | 2 | -0.70 (-2.42, 1.01) | 0.421 | 0.384 | 0.0% | 0.196 |  |
| Obese (>30) | 3 | 0.93 (-0.86, 2.73) | 0.309 | 0.743 | 0.0% |  |  |
| Subgroup analyses of carnitine on DBP (mmHg) | | | | | | | |
| Overall effect | 5 | -0.67 (-1.82, 0.47) | 0.251 | 0.239 | 27.3% |  |  |
| Baseline BMI (kg/m^2^) |  |  |  |  |  |  |  |
| Overweight (25-29.9) | 2 | 0.56 (-3.09, 4.23) | 0.762 | 0.067 | 70.2% | 0.464 |  |
| Obese (>30) | 3 | -0.86 (-1.98, 0.25) | 0.129 | 0.419 | 0.0% |  |  |
| Subgroup analyses of carnitine on serum CRP (mg/dl) | | | | | | | |
| Overall effect | 4 | -0.07 (-0.13, -0.01) | **0.037** | 0.004 | 77.2% |  |  |
| Trial duration (week) |  |  |  |  |  |  |  |
| ˂12 | 2 | -0.30 (-0.45, -0.14) | **<0.001** | 0.619 | 0.0% | **0.001** |  |
| ≥12 | 2 | -0.03 (-0.06, -0.01) | **0.024** | 0.175 | 45.8% |  |  |
| Subgroup analyses of carnitine on serum TNF-α (pg/ml) | | | | | | | |
| Overall effect | 3 | -1.39 (-2.67, -0.11) | **0.033** | <0.001 | 97.1% |  |  |
| Subgroup analyses of carnitine on Weight (Kg) | | | | | | | |
| Overall effect | 6 | -1.58 (-2.53, -0.63) | **0.001** | 0.718 | 0.0% |  |  |
| Trial duration (week) |  |  |  |  |  |  |  |
| ˂12 | 2 | -1.91 (-4.30, 0.47) | 0.116 | 0.831 | 0.0% | 0.767 |  |
| ≥12 | 4 | -1.52 (-2.55, -0.48) | **0.004** | 0.432 | 0.0% |  |  |
| Baseline BMI (kg/m^2^) |  |  |  |  |  |  |  |
| Overweight (25-29.9) | 2 | -2.42 (-5.34, 0.49) | 0.104 | 0.735 | 0.0% | 0.552 |  |
| Obese (>30) | 4 | -1.48 (-2.48, -0.48) | **0.004** | 0.491 | 0.0% |  |  |
| Subgroup analyses of carnitine on BMI (kg/m^2^) | | | | | | | |
| Overall effect | 14 | -0.28 (-0.51, -0.05) | **0.017** | 0.037 | 44.4% |  |  |
| Trial duration (week) |  |  |  |  |  |  |  |
| ˂12 | 4 | -0.86 (-1.68, -0.03) | **0.041** | 0.941 | 0.0% | 0.159 |  |
| ≥12 | 10 | -0.24 (-0.48, 0.01) | 0.051 | 0.026 | 52.5% |  |  |
| Baseline BMI (kg/m^2^) |  |  |  |  |  |  |  |
| Overweight (25-29.9) | 8 | -0.17 (-0.45, 0.10) | 0.230 | 0.507 | 0.0% | 0.420 |  |
| Obese (>30) | 6 | -0.37 (-0.77, 0.02) | 0.069 | 0.007 | 68.5% |  |  |
| Subgroup analyses of carnitine on BFP (%) | | | | | | | |
| Overall effect | 3 | -1.83 (-2.70, -0.95) | **<0.001** | 0.347 | 5.5% |  |  |
| Subgroup analyses of carnitine on Leptin (ng/ml) | | | | | | | |
| Overall effect | 3 | -2.21 (-3.67, -0.75) | **0.003** | 0.175 | 42.5% |  |  |
| Subgroup analyses of carnitine on Apo A (mg/dl) | | | | | | | |
| Overall effect | 6 | -0.48 (-9.14, 8.18) | 0.913 | <0.001 | 91.9% |  |  |
| Subgroup analyses of carnitine on Apo B (mg/dl) | | | | | | | |
| Overall effect | 7 | -7.66 (-20.91 , 5.58) | 0.257 | <0.001 | 96.8% |  |  |

Abbreviations: Apo A, Apolipoprotein A; Apo B, Apolipoprotein B; BFP, body fat percentage; BMI, body mass index; CI, confidence interval; CRP, c-reactive protein; FBG, fasting blood glucose; HbA1c, hemoglobin A1c; HDL, high-density lipoprotein; HOMA-IR, homeostatic model assessment for insulin resistance; LDL, low-density lipoprotein; DBP, diastolic blood pressure; SBP, systolic blood pressure; TC, total cholesterol, TG, triglyceride; TNF-α, tumor necrosis factor alpha; WMD, weighted mean differences.
