# Supplementary material for: The effects of L-carnitine supplementation on cardiovascular risk factors in participants with impaired glucose tolerance and diabetes: a systematic review and dose–response meta-analysis
Source: Diabetol Metab Syndr. 2024 Jul 31;16:185. doi: 10.1186/s13098-024-01415-8 (PMC11290177; doi:10.1186/s13098-024-01415-8)
Supplement: Supplementary file 3 — Supplementary Material 3. [file 13098_2024_1415_MOESM3_ESM.docx]

**Supplementary Table 3.** GRADE profile of L-carnitine for CVD risk factors in adults.

| **Outcomes** | **Risk of bias** | **Inconsistency** | **Indirectness** | **Imprecision** | **Publication Bias** | **WMD (95%CI)** | **Quality**  **of evidence** |
| --- | --- | --- | --- | --- | --- | --- | --- |
| TG | Serious limitation ^1^ | Very serious limitation ^2^ | No serious limitation | No serious limitation | No serious limitation | -13.50 (-26.33, -0.67) | ⊕◯◯◯  Low |
| TC | Serious limitation ^1^ | Very serious limitation ^2^ | No serious limitation | Serious limitation ^3^ | No serious limitation | -6.49 (-21.93, 8.93) | ◯◯◯◯  Very low |
| LDL | Serious limitation ^1^ | Very serious limitation ^2^ | No serious limitation | No serious limitation | No serious limitation | -12.66 (-18.12, -7.21) | ⊕◯◯◯  Low |
| HDL | Serious limitation ^1^ | Very serious limitation ^2^ | No serious limitation | Serious limitation | No serious limitation | 1.07 (-1.13, 3.28) | ◯◯◯◯  Very low |
| FBG | Serious limitation ^1^ | Very serious limitation ^2^ | No serious limitation | No serious limitation | No serious limitation | -6.24 (-9.80, -2.69) | ⊕◯◯◯  Low |
| Insulin | No serious limitation | Very serious limitation ^2^ | No serious limitation | Serious limitation | No serious limitation | -1.131 (-2.52, 0.26) | ⊕◯◯◯  Low |
| HbA1C | No serious limitation | Very serious limitation ^2^ | No serious limitation | No serious limitation | No serious limitation | -0.37 (-0.67, -0.07) | ⊕⊕◯◯  Moderate |
| HOMA-IR | No serious limitation | Very serious limitation ^2^ | No serious limitation | No serious limitation | No serious limitation | -0.72 (-1.40, -0.04) | ⊕⊕◯◯  Moderate |
| SBP | No serious limitation | No serious limitation | No serious limitation | Serious limitation | No serious limitation | 0.07 (-1.16, 1.32) | ⊕⊕⊕◯  High |
| DBP | No serious limitation | No serious limitation | No serious limitation | Serious limitation | No serious limitation | -0.67 (-1.82, 0.47) | ⊕⊕⊕◯  High |
| CRP | No serious limitation | Very serious limitation ^2^ | No serious limitation | No serious limitation | No serious limitation | -0.07 (-0.13, -0.01) | ⊕⊕◯◯  Moderate |
| TNF-α | No serious limitation | Very serious limitation ^2^ | No serious limitation | No serious limitation | No serious limitation | -1.39 (-2.67, -0.11) | ⊕⊕◯◯  Moderate |
| Weight | No serious limitation | No serious limitation | No serious limitation | No serious limitation | No serious limitation | -1.58 (-2.53, -0.63) | ⊕⊕⊕⊕  Very high |
| BMI | No serious limitation | No serious limitation | No serious limitation | No serious limitation | No serious limitation | -0.28 (-0.51, -0.05) | ⊕⊕⊕⊕  Very high |
| BFP | No serious limitation | No serious limitation | No serious limitation | No serious limitation | No serious limitation | -  1.83 (-2.70, -0.95) | ⊕⊕⊕⊕  Very high |
| Leptin | No serious limitation | No serious limitation | No serious limitation | No serious limitation | No serious limitation | -2.21 (-3.67, -0.75) | ⊕⊕⊕⊕  Very high |
| Apo A | Serious limitation ^1^ | Very serious limitation ^2^ | No serious limitation | Serious limitation | No serious limitation | -0.48 (-9.14, 8.18) | ⊕◯◯◯  Low |
| Apo B | Serious limitation ^1^ | Very serious limitation ^2^ | No serious limitation | Serious limitation | No serious limitation | -7.66 (-20.91 , 5.58) | ◯◯◯◯  Very low |

1. General risk of bias is bad for > 50% of included studies.
2. There is significant heterogeneity for TG (I^2^=97.3%), TC (I^2^=98.3%), LDL (I^2^=91.5%), HDL (I^2^=96.2%), FBG (I^2^=91.0%), Insulin (I^2^=88.4%), HbA1C (I^2^=92.6%), HOMA-IR (I^2^=91.0%), CRP (I^2^=77.2%), TNF-α (I^2^=97.1%), Apo A (I^2^=91.9%), and Apo B (I^2^=96.8%).
3. There is no evidence of significant effects of L-carnitine supplementation on TC, HDL, Insulin, SBP, DBP, Apo A, and Apo B.
